# Revealing the Causal Relationship Between Differential White Blood Cell Counts and Depression: A Bidirectional Two-Sample Mendelian Randomization Study

**DOI:** 10.1155/da/3131579

**Published:** 2025-03-03

**Authors:** Ying Cao, Xuguang Li, Jing Gao, Nan Zhang, Guoqian Zhang, Shen Li

**Affiliations:** ^1^Department of Clinical Laboratory, First Teaching Hospital of Tianjin University of Traditional Chinese Medicine, Tianjin, China; ^2^National Clinical Research Center for Chinese Medicine Acupuncture and Moxibustion, Tianjin, China; ^3^Health Care Center, Institute of Radiation Medicine, Chinese Academy of Medical Sciences and Peking Union Medical College, Tianjin, China; ^4^Institute of Mental Health, Tianjin Anding Hospital, Mental Health Center of Tianjin University, Tianjin, China; ^5^Brain Assessment and Intervention Laboratory, Tianjin Anding Hospital, Mental Health Center of Tianjin University, Tianjin, China

**Keywords:** depression, finnGen, Mendelian randomization, white blood cells

## Abstract

**Background:** The link between white blood cells (WBC) and depression has been studied, but the causal relationship remains unclear. This study aimed to elucidate the potential bidirectional causal links between six specific WBC count features and depression using a two-sample Mendelian randomization (MR) analysis, leveraging summary statistics from genome-wide association studies (GWAS).

**Method:** The dataset on depression (*N* = 406,986) was sourced from the FinnGen database, while the dataset on WBC (*N* = 563,085) was obtained from a combined dataset of Blood Cell Consortium (BCX) and UK Biobank. The MR analyses employed include inverse variance weighted (IVW), MR-Egger, weighted median, contamination mixture method (conmix), and constrained maximum likelihood-based Mendelian randomization (cML-MA). A threshold *p* < 0.05 after false discovery rate (FDR) correction was set as the criterion for causality based on IVW.

**Results:** Reverse MR analysis indicated a causal relationship where depression leads to an increase in overall WBC count (IVW beta = 0.031, *p* = 0.015, *p*_FDR_ = 0.044) and specifically in basophil count (IVW beta = 0.038, *p* = 0.006, *p*_FDR_ = 0.038), with a marginally significant impact on lymphocyte count (beta = 0.029, *p* = 0.036, *p*_FDR_ = 0.071). Furthermore, forward MR analysis suggested a potential role of monocyte count in decreasing depression risk (*p* = 0.028), though this association did not retain statistical significance after FDR correction.

**Conclusion:** These findings suggest that depression may causally influence the immune system by elevating overall WBC and basophil counts, with a marginally significant increase in lymphocyte levels. Conversely, higher monocyte count might confer some protection against depression, albeit with less statistial certainty. This study provides novel insights into the complex interplay between depression and immune function.

## 1. Introduction

Depression, a prevalent mental disorder, affects over 300 million individuals worldwide, accounting for approximately 4%–5% of the global population, as reported by the World Health Organization [1]. Characterized by negative affect, sleep disturbances, psychomotor retardation, impaired cognition, and potential suicidal ideation [2–4], depression poses a substantial burden on healthcare systems [5] and socioeconomic structures [6]. Despite its widespread impact [7], the pathological and physiological mechanisms underlying depression remain only partially understood.

Elevated levels of white blood cells (WBC) [8], C-reactive protein [9], and both peripheral and central inflammatory cytokines, such as IL-6 [10, 11], the proinflammatory protein tumor necrosis factor-*α* (TNF*α*) [12, 13], and monocyte chemoattractant protein (MCP)-1/CCL2 [14] have been identified in patients with depression, suggesting a potential link between inflammation and depressive symptomatology. The association between elevated peripheral inflammatory markers (PIM) and depressive symptoms has garnered attention [15, 16]. The WBC count, an indicator of low-grade inflammation, reflects the overall activity of immune system. Although, several studies have indicated a correlation between peripheral WBCs and depression [17–20], the specific role of WBC subtypes in depression is still being explored.

Neutrophils, as the predominant subtype of WBCs, have been implicated in heightened psychological stress [21]and neuroinflammation [22] associated with depression. While certain studies indicate elevated neutrophil counts [23] and an increased neutrophil-to-lymphocyte ratio (NLR) [24, 25] in patients with depression, correlating with the severity of symptoms, other studies present inconsistent findings [26]. Furthermore, it remains uncertain whether alterations in neutrophil levels are causative factors or merely consequences within the pathophysiology of depression.

Monocytes, precursors to tissue macrophages and dendritic cells, are involved in depression through cytokine production [27–29] and chemotaxis [30], correlating with symptom severity [23]. Some studies have reported increased monocyte counts in depressed patients [23], while some others find no significant link between monocyte activity and the clinical progression of depression [31].

Recent studies on lymphocytes in depression have yielded conflicting results. Although some studies indicate decreased lymphocyte counts in depressive patients [17, 32, 33], other research has found that increased T-cell subset counts (particularly CD8+ cells) correlate with depression severity [34]. Evidence suggests impaired lymphocyte proliferation and decreased natural killer cell activity in major depressive disorder (MDD) patients [35]. The relationship between lymphocyte function and depression remains controversial [36, 37], with debates centered on whether immune activation or suppression predominates [38, 39].

These discrepancies may be primarily attributed to the susceptibility of conventional observational studies to confounding factors, selection bias [40, 41], and the difficulty of excluding reverse causality. Consequently, comprehensive investigations into the relationship and mechanisms between WBC and depression have yet to be fully explored.

Mendelian randomization (MR) analysis emerges as a groundbreaking tool in epidemiology, harnessing genetic variation to deduce causal relationships between modifiable risk factors and health outcomes. Based on genetic variation [42], this innovative approach leverages natural random allocation of genotypes to establish causal relationships between exposures and outcomes. A key strength of MR lies in its resilience against confounding, a common pitfall in traditional observational studies. Additionally, MR is less prone to reverse causation, as genetic variants precede the onset of disease [43]. The application of a bidirectional two-sample MR analysis offers a novel and powerful approach to untangle the complex relationship between peripheral WBC counts and depression.

To the best of our knowledge, this represents the first MR study investigating WBC count traits and depression. Previous research by Perry et al. [44], while examining inflammatory markers and psychiatric disorders, was restricted to neutrophils and lymphocytes without bidirectional analysis. Our study advances this field through comprehensive bidirectional MR analyses across WBC subtype count traits and depression. Employing a methodologically robust framework that incorporates contemporary MR approaches and extensive sensitivity testing, we provide compelling evidence for causal relationships between WBC traits and depression. This thorough investigation not only deepens our understanding of immune-psychiatric pathways but also offers promising insights into depression pathophysiology, potentially unveiling novel therapeutic targets and treatment strategies.

## 2. Materials and Methods

### 2.1. Study Design

**Figure 1:**
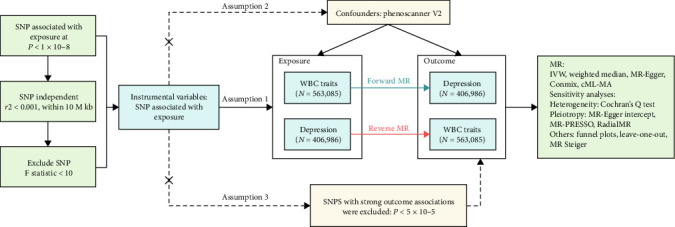
Overview of the MR analysis process. This flowchart illustrates the selection and validation of instrumental variables for assessing the causal relationship between WBC traits and depression. The analysis adheres to three core MR assumptions: Workflow of the current study and fundamental assumptions of MR analysis: The genetic instruments should (a) exhibit an association with the exposure, (b) remain unaffected by confounding factors, and (c) be independent of the outcome. Forward MR examines the effect of WBC traits on depression, while reverse MR assesses the impact of depression on WBC traits. MR: Mendelian randomization; WBC: white blood cell; SNP: single nucleotide polymorphism.

We conducted a systematic analysis to establish the causal impact of peripheral WBC counts on the depression risk (see Figure 1).Our MR analysis framework commenced with rigorous instrumental variable (IV) selection, adhering to three fundamental MR assumptions [45, 46]: (1) the association assumption, which necessitates that IVs demonstrate robust associations with exposure factors; (2) the independence assumption, which requires that IVs are not associated with potential confounders; and (3) the exclusion restriction assumption, which posits that genetic variations solely influence outcomes through exposure factors rather than through alternative pathways. Leveraging comprehensive GWAS data from both white blood cell traits and depression cohorts, we conducted bidirectional MR analyses to evaluate potential causal relationships in both directions. The statistical framework incorporated multiple analytical approaches, supplemented by extensive sensitivity analyses, to establish robust causal inference while effectively mitigating potential biases and confounding factors. This methodologically rigorous approach provides a comprehensive foundation for elucidating the causal relationships between the investigated phenotypes. The findings of this study have been documented in accordance with the STROBE-MR guidelines [47, 48] (Supporting Information 1: Table S1).

### 2.2. Data

#### 2.2.1. Genome-Wide Association Study Data on WBC Counts

The genome-wide association studies (GWAS) data for circulating WBC counts in this study were derived from the comprehensive analysis conducted by Chen et al. and Vuckovic et al. [49, 50]. This meta-analysis represents the most extensive and contemporary GWAS of hematological traits, encompassing the largest sample size to date. This pivotal research conducted an extensive whole-genome discovery analysis within a cohort of 408,112 individuals of European ancestry, sourced from the UK Biobank. It further extended genetic association testing to an additional cohort of 154,973 participants of European descent, affiliated with the Blood Cell Consortium (BCX). The analysis covered six distinct features of circulating blood cell counts, namely basophil cell count, white blood cell count, monocyte cell count, lymphocyte cell count, eosinophil cell count, and neutrophil cell count. For detailed methodology and results, please refer to the literature (PMID: 32888494, 32888493).

#### 2.2.2. Genome-Wide Association Study Data on Depression

To eliminate the risk of sample overlap, depression data from the UK Biobank were intentionally omitted from our study. Instead, the FinnGen database was selected as the primary data source [51], owing to its genetically homogeneous population structure and robust nationwide healthcare registry system, which collectively minimize population stratification bias while ensuring standardized phenotyping. The FinnGen project is an extensive genomics initiative that has analyzed over 500,000 samples from the Finnish biobank, with the objective of collecting and analyzing genetic, medical, and lifestyle data to gain insights into disease mechanisms and susceptibilities. Within the chosen dataset from FinnGen, depression cases (*N* = 47,696) were defined based on diagnoses of single depressive episodes (ICD-10 F32, including mild, moderate, or severe depressive episodes), recurrent depressive disorder (ICD-10 F33), psychotic depression (ICD-9 296.1|296.8|300.4), and other forms of MDD, with a mean age of first onset at 41.76 years. The controls (*N* = 359,290) excluded individuals with any mood disorders, specifically mania, bipolar disorder, persistent mood disturbances, and other mood disorders, including those cases where the severity or duration was insufficient to meet the diagnostic criteria specified in ICD10 F30-F34.

This investigation entailed a reanalysis of publicly available data previously collected and documented, thereby obviating the need for additional ethical approval.

### 2.3. Method

#### 2.3.1. Instrumental Variable Selection

The selection of IVs is predicated upon the three fundamental assumptions of MR previously elucidated. In this study, we identified single nucleotide polymorphisms (SNPs) associated with circulating leukocyte counts and its subtypes from GWAS data, employing a stringent screening criterion of *p* < 5 × 10^−8^. To ensure the independence of each SNP, we excluded those in linkage disequilibrium (*r*^2^ <0.001) and located more than 10,000 kb apart physically between genes. Subsequently, we calculated F-statistics [52], crucial for evaluating weak instrument bias—an F-statistics less than 10 indicating potential weak instrument bias [53]—and omitted such SNPs to safeguard against bias in our findings. This threshold ensures sufficient instrument strength for reliable causal estimation. The F-statistic is derived from the formula:

(1) $$\begin{array}{c} F=\frac{R^{2}\times \left(n-k-1\right)}{k\times \left(1-R^{2}\right)}, \end{array}$$

where *n* represents the sample size, *k* represents the number of IVs used, and *R*^2^ reflects the extent to which IVs explain exposure [54]. The calculation formula for *R*^2^ is defined as

(2) $$\begin{array}{c} R^{2}=2\times \left(1-\text{MAF}\right)\times \text{MAF}\times \beta^{2}, \end{array}$$

where MAF denotes the minimum allele frequency, and *β* represents the effect value of the allele.

To minimize potential pleiotropy, SNPs highly correlated with outcomes (*p* < 5 × 10^−5^), along with all palindromic SNPs, were excluded during instrumental variable selection to ensure the harmonization of exposure and outcome, grounded on the assumption of independence. To uphold the exclusion restriction assumption, phenotypes corresponding to IVs underwent scrutiny in the phenoscanner database [55, 56] (http://www.phenoscanner.medschl.cam.ac.uk/) to exclude potential confounding SNPs that might influence outcomes. Factors associated with depression, such as type 2 diabetes [57], Crohn's disease [58], tumors [59], cardiovascular diseases [60], and obesity [61], were excluded based on an exhaustive literature review. This step minimizes potential violation of the exclusion restriction assumption.

For statistical validation, outliers were identified and removed using MR_PRESSO test [62] and Radial MR [63]. Additionally, SNPs exhibiting incorrect causal directions were excluded using MR Steiger [64], resulting in a final set of IVs. These statistical approaches ensure the robustness of our genetic instruments.

The identical methodology described above was applied for extracting IVs associated with depression in reverse MR analysis, maintaining consistency in our analytical approach.

#### 2.3.2. MR Analysis

The analyses were executed utilizing the TwoSampleMR (version 0.5.10), MR (version 0.8.0), MRPRESSO package (version 1.0), and RadialMR package in R Software (version 4.3.2). Five different MR methods were employed for analysis, including the traditional IVW [65, 66], weighted median [67], MR-Egger [68], conmix [69], and cML-MA [70, 71]. The IVW method served as the cornerstone of our MR analysis, providing reliable causal estimates under the assumption of no directional pleiotropy [65, 66]. The weighted median approach, requiring at least 50% valid IVs, aimed to mitigate potential biases by accommodating the effects of horizontal pleiotropy, thereby providing a reliable causality estimate. The MR-Egger regression method was also used to assess the robustness of the IVW results, particularly against the backdrop of potential assumption violations. The conmix method distinguished itself by aggregating genetic variants that share similar causal estimates, thus facilitating a nuanced exploration of risk factor mechanisms and their impact on outcomes [69]. In response to challenges posed by pleiotropy and confounding variables hidden among IVs, we employed cML-MA, a method combining constrained maximum likelihood with model averaging, offering superior power and reliability over MR-Egger in detecting pleiotropic effects.

Sensitivity analyses encompassed several techniques: Cochran's Q test assessed heterogeneity among SNP estimates, the MR-Egger intercept [68], and MR-PRESSO method [62] for identifying horizontal pleiotropy. Outliers pinpointed by the MR-PRESSO method were removed, refining the assessment of causal relationships. Persistent horizontal pleiotropy cases were further scrutinized using the Radial MR approach [63] to eliminate outliers and reassess the causal inference.

Furthermore, visual aids such as scatter plots, the “leave-one-out” strategy, and funnel charts were employed for a comprehensive examination of the exposure–outcome relationship. Scatter plots were particularly useful in determining the nature of the exposure as either a risk or protective factor. The “leave-one-out” approach allowed for the assessment of individual SNP contributions to the overall causal relationship, enhancing the robustness of our analysis. Funnel charts aided in identifying heterogeneity and outliers, offering a visual testament to the reliability of the correlations. For binary outcomes, odds ratios (ORs) along with their 95% confidence intervals (CIs) were computed to gauge the strength of causality; significance was determined through multiple testing, with adjusted *p*-values <0.05 after false discovery rate (FDR) correction denoting statistical significance.

## 3. Result

### 3.1. Exploring the Causal Effect for WBC Count Features on Depression

Initially, a total of 201, 507, 510, 510, 448, and 428 SNPs associated with basophil cell count, white blood cell count, monocyte cell count, lymphocyte cell count, eosinophil cell count, and neutrophil cell count, respectively, were included (Supporting Information 2: Table S2). Following depression, data matching, and the removal of redundant SNPs (Supporting Information 3: Table S3), we meticulously cross-referenced phenotypes corresponding to the IVs in the Phenoscanner database (Supporting Information 4: Table S4), simultaneously excluding any potentially confounding SNPs. Outliers were detected and removed using MR_PRESSO and Radial MR methods (Supporting Information 5: Table S5 and Supporting Information 6: Figure S1), with subsequent application of the MR Steiger test to eliminate SNPs with incorrect causal direction. Ultimately, we obtained a set of 164, 391, 411, 400, 352, and 328 IVs, respectively (Supporting Information 7: Table S6), each accompanied by F-statistics ranging from 11.2 to 3998.61, indicating minimal susceptibility to weak instrument bias.

**Figure 2:**
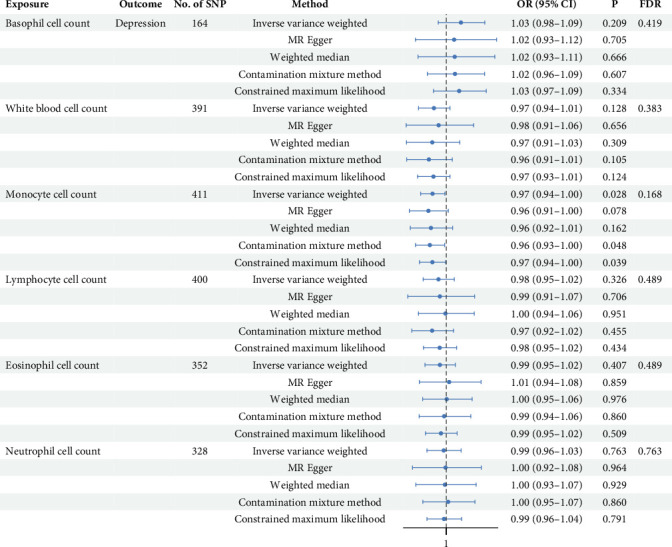
Forest plot of MR estimates for the causal effect of WBC subtypes on depression risk. This figure presents the results of MR analyses examining the causal relationship between six WBC subtypes and depression. ORs with 95% CIs are plotted on a logarithmic scale, with the vertical dashed line representing no effect (OR = 1).CI = confidence intervals; FDR = false discovery rate; IVW = inverse variance weighted; MR = Mendelian randomization; OR = odds ratios; SNP = single nucleotide polymorphism; WBC: white blood cell.

Employing five MR methods, the results obtained from the IVW method serve as the primary criterion for assessment (see Figure 2). The results for monocytes counts in relation to depression were as follows: IVW OR = 0.97, with a 95% CI of 0.94–1.00, *p* = 0.028, *p*_FDR_ = 0.168. The findings from the other MR methods consistently supported the directionality observed in IVW analysis. Although the statistical significance did not persist following FDR correction, the consistent directionality observed across other MR methods suggests potential implications deserving of further validation through larger sample sizes. Additionally, no evidence of a causal effect of WBC counts or other subtypes on depression was found (Supporting Information 8: Table S7 and Supporting Information 9: Figure S2).

### 3.2. Exploring the Causal Effect for Depression on WBC Count

**Figure 3:**
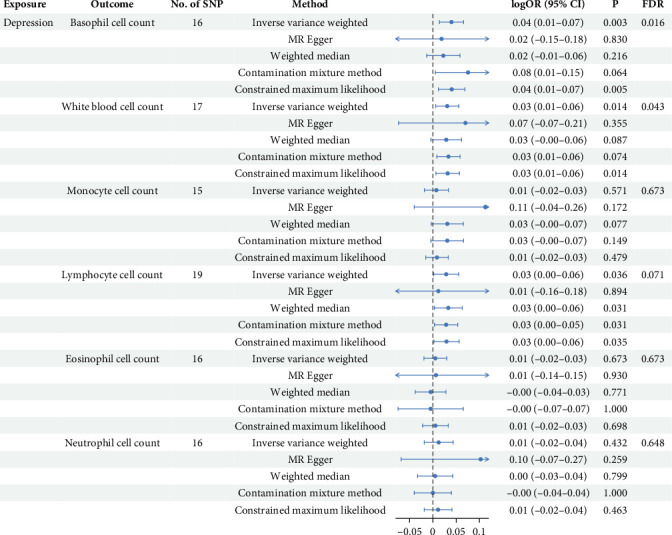
Forest plot of MR estimates for the causal effect of depression on WBC subtypes. This figure presents the results of a multi-method MR analysis investigating the potential causal influence of depression on various white blood cell counts. The *x*-axis represents the logOR with 95% CI. The vertical dashed line at *x* = 0 indicates no effect. CI, confidence intervals; FDR: false discovery rate; IVW, inverse variance weighted; MR, Mendelian randomization; OR, odds ratios; SNP, single nucleotide polymorphism.

In the reverse MR analysis focusing on depression, a total of 22 SNPs associated with depression were identified and extracted (Supporting Information 10: Table S8). After matching with WBC count, removal of palindrome structures (Supporting Information 11: Table S9), exclusion of outliers (Supporting Information 12: Table S10 and Supporting Information 13: Table S11), and elimination of SNPs with incorrect causal directions, we derived a final set of IVs for subsequent MR analysis. Specifically, we obtained 16 IVs for basophil cell count, 17 for WBC count, 15 for monocyte cell count, 19 for lymphocyte cell count, 16 for eosinophil cell count, and 17 for neutrophil cell count, respectively (Supporting Information 14: Table S12). The F-statistic ranged from 309.23 to 603.91, indicating minimal susceptibility to weak instrument bias. As shown in Figure 3, the findings demonstrated a significant positive correlation between depression and WBC count (IVW beta = 0.031, *p* = 0.015, *p*_FDR_ = 0.044), which aligned with the directional consistency observed in other analytical approaches' beta values. Similarly, a notable positive association was observed between depression and basophil cell count (IVW beta = 0.038, *p* = 0.003, *p*_FDR_ = 0.016), affirming the results obtained from alternative methods. Furthermore, a marginal significance was noted in the positive correlation between depression and lymphocyte levels (IVW beta = 0.029, *p* = 0.036, *p*_FDR_ = 0.071). The figure illustrates the outcomes of other methodologies that exhibit concordant trends with those derived from IVW analysis (Supporting Information 15: Table S13 and Supporting Information 16: Figure S3).

### 3.3. Sensitivity Analysis

Sensitivity analysis was undertaken to evaluate the heterogeneity of the data. The Cochran's Q test (Q_pval >0.05) indicated no significant evidence of heterogeneity (Supporting Information 17: Table S14 and Supporting Information 18: Table S15). Additionally, the MR-Egger intercept was calculated. (Supporting Information 19: Table S16 and Supporting Information 20: Table S17). The egger_intercept values for both forward MR (0.0071–0.0012) and reverse MR (0.0035–0.0042) were all below 0.01, with all *p*-values exceeding 0.05, suggesting the absence of directional pleiotropy. MR_PRESSO analysis (Supporting Information 21: Table S18 and Supporting Information 22: Table S19) also revealed that all Global Test *p*-values were above 0.05, indicating no detection of horizontal pleiotropy. Furthermore, no evidence of heterogeneity was observed in the funnel plot analysis (Supporting Information 23: Figure S4 and Supporting Information 24: Figure S5). Moreover, leave-one-out analysis demonstrated the robustness of the observed causal relationship (Supporting Information 25: Figure S6 and Supporting Information 26: Figure S7).

## 4. Discussion

This study marks the inaugural utilization of a large-scale GWAS dataset, employing a two-sample MR method to assess the causal relationship between six WBC traits and susceptibility to depression. In the forward MR analysis, while monocytes showed suggestive evidence of reducing depression risk, insufficient support currently exists for an association between the six white blood cell count features and depression susceptibility. Conversely, reverse MR results indicate that depression may lead to increased levels of WBC counts and basophil granulocyte counts. Furthermore, marginal significance was observed in raising lymphocyte counts statistically. The findings tentatively suggest that monocyte count may hold suggestive significance in mitigating the risk of depression. However, contrasting results from a meta-analysis [72] and observational study [23] indicate heightened levels of monocytes in patients with depression. Interestingly, mechanistic studies [73] heightened activity in gene clusters associated with apoptosis/growth and lipid/cholesterol pathways in monocytes derived from individuals with depression, suggesting cellular senescence, mitochondrial apoptotic dysfunction, and aberrant inflammatory processes. Additionally, studies [30, 74] have underscored the critical role of monocyte recruitment to the brain in depression development. Hence, we postulate that the monocyte–macrophage system plays a pivotal role in the depression pathogenesis, and augmenting monocyte count may hold potential therapeutic promise in mitigating depression risk. However, the observed elevation of monocytes in depressed patients could be attributed to compensatory mechanisms or confounding factors. Further research is imperative to validate this hypothesis.

Moreover, the study findings indicate a unidirectional causal relationship between depression and WBC, with depression leading to an increase in WBC count. A meta-analysis [75] of 104 studies on immune cell composition in MDD over the past two decades also found that patients with depression had significantly higher levels of WBC. While this aligns with our study's results, it does not establish causality between these factors. WBC count can serve as an indicator of overall immune system activity. Currently, scholars propose two models for the interaction between depression and inflammatory states: the neuroinflammation model [76] and stress response model [77]. The former suggests that activation of the immune system increases risk for depression, while chronic inflammation is closely associated with depressive episodes and progression. In contrast, the latter posits that depression may accelerate inflammatory responses, leading to changes in WBC quantity and function—representing a stress-induced proinflammatory model. Some argue [16] that both models coexist to form a feedback loop. From our perspective, following principles from the stress response model, depression elevates levels of WBC.

The results revealed no discernible causal effect of lymphocytes on depression. However, in the reverse MR analysis, a trend emerged indicating that depression might lead to an increase in lymphocyte levels, albeit with only marginal statistical significance. Our findings both complement and contrast with existing literature. While some studies have reported a significant decrease in lymphocyte count among patients with depression [78], others demonstrate positive correlations between lymphocyte counts and depression severities [79]. This apparent contradiction might be explained by the complex immunomodulatory mechanisms involved. Recent mechanistic studies have revealed that lymphocytes play a role in regulating neurotransmitter release and neuronal activity, through multiple pathways, including cytokine production and direct cellular interactions [80]. These diverse mechanisms might contribute to the heterogeneous findings across studies.

The findings of this study suggest a unidirectional causal relationship between depression and basophil granulocyte count, implying that depression may lead to increased levels of basophil granulocytes. This novel finding represents a significant advancement in our understanding of the immune–depression relationship. Currently, limited research has been conducted on the association between basophil granulocytes and depression. In a study by Baek et al. [81], anxiety levels were assessed using the Hamilton Depression Rating Scale among 709 newly diagnosed patients with MDD, revealing a significant negative correlation between basophil granulocyte count and the severity of depressive anxiety. However, this specific study did not directly investigate the connection between basophil granulocytes and depression. Our findings extend beyond previous research by establishing, for the first time, a potential causal relationship between depression and elevated basophil granulocyte count. This discovery opens new avenues for investigation into the biological mechanisms underlying depression and suggests potential therapeutic targets.

Regarding eosinophil count, this study did not find a causal relationship between it and depression. Lin et al. [82] conducted an analysis of NHANES 2007–2014 data and developed a predictive model—utilizing blood biomarkers to predict depression as well as depression coexisting with diabetes, obesity, or metabolic syndrome. The results showed that in the models for patients with diabetes and metabolic syndrome, eosinophil count and percentage were among the most important features. Moreover, Steel et al. [83] observed a positive correlation between the degree of weakness and depression levels in patients with liver cancer. However, Singh et al. [84] noted reduced eosinophil counts among patients diagnosed with MDD exhibited, with treatment resulting in normalized eosinophil counts among MDD patients initially experiencing severe depression. The interaction between eosinophils and other factors may exert an influence on depression. The current state of research suggests that the understanding of the association between eosinophils and depression remains incomplete.

Furthermore, the research findings of this article indicate no causative association between neutrophils and depression. These results are consistent with Vulser et al. [85], who found no significant association between depressive symptoms and neutrophil count after controlling for potential confounding factors [85]. However, it is important to note that other studies [23] have reported an increase in neutrophil count and abnormal activity in patients with depression, indicating a potential correlation with the onset and progression of depression [86]. Specifically, among individuals with depression of different genders, female patients tend to exhibit elevated levels of neutrophils accompanied by decreased lymphocyte counts [87]. Conversely, some research suggests that inflammatory markers may predict depression in males but not females [88, 89]. Furthermore, it has been proposed that the observed elevation in white blood cell count and increased levels of neutrophils in depressed patients could be attributed to medication use as a confounding factor [90]. Therefore, caution should be exercised when interpreting the association between depression and neutrophils in observational studies due to potential confounders.

This study has several noteworthy strengths. First, it pioneers the application of a dual-sample MR method to comprehensively evaluate the causal relationship between six leukocyte characteristics and susceptibility to depression. Previous research by Perry et al. [44] utilized MR analysis to investigate the causal relationship between lymphocyte and neutrophil counts and depression; however, they were unable to establish bidirectional causality. Our study represents a substantial advancement over prior investigations, encompassing a significantly larger sample size—approximately 3.3 times greater than its predecessor's—thereby providing enhanced statistical power and reliability. Our study results mutually validate and complement those of previous research endeavors in this domain. While our findings regarding neutrophils align with previous studies, we uniquely identified a potential marginal increase in lymphocyte count levels associated with depression. Furthermore, our comprehensive evaluation of six WBC traits provides a more complete picture of immune system involvement in depression compared to earlier studies that focused on limited cell types. This broader scope enables a more nuanced understanding of the complex relationship between immune function and depression. Second, apart from employing traditional methods such as IVW, MR-Egger, and weighted median analyses in MR analysis, we employed Conmix—a method proficient at handling large quantities of IVs—as well as newer approaches like cML-MA. The consistent confirmation of findings across these different methods bolsters the persuasiveness of the identified causal relationships.

However, this study also faces several limitations. A primary limitation of this study lies in the exclusive utilization of depression data from the FinnGen consortium. While this approach effectively prevented sample overlap with UK Biobank WBC data, it introduces potential constraints on the generalizability of our findings. Although the Finnish population's genetic homogeneity offers methodological advantages, it may not comprehensively capture the genetic and environmental heterogeneity present across European populations. Consequently, future validation studies encompassing diverse population cohorts are essential to evaluate the broader applicability of these genetic associations. Second, this study solely addresses causal relationships and does not address linear relationships wherein the severity of depressive symptoms correlates with higher or lower counts of WBC or its subtypes. Lastly, it should be noted that current evidence is limited to European populations due to potential heterogeneity among different racial groups. Therefore, these findings are only generalizable to European populations.

## 5. Conclusions

In conclusion, these findings offer novel insights into the immune-inflammatory hypothesis of depression. However, it is essential to acknowledge that these conclusions should be regarded as preliminary speculations, offering a noteworthy perspective for exploring risk factors related to depression and inspiring future clinical practices and interventions. Nevertheless, prior to their implementation in practical clinical settings, it is imperative to meticulously consider their limitations and cautiously evaluate their reliability and applicability.

## Data Availability

Summary statistics for basophil cell count, white blood cell count, monocyte cell count, lymphocyte cell count, eosinophil cell count, and neutrophil cell count are available on the IEU OpenGWAS project (https://gwas.mrcieu.ac.uk/) with GWAS IDs ieu-b-29 to ieu-b-34 respectively. The depression data utilized in this study were sourced from the FinnGen database documentation of R10 release (https://finngen.gitbook.io/documentation/data-download); The phenocode was “F5_DEPRESSIO,” and the phenotype was “Depression.” The Supporting Information 27: Table S20 provides further information.

## Supporting Information

Supporting Information 1 Table S1: STROBE-MR checklist.

Supporting Information 2 Table S2: Extract IVs for WBC.

Supporting Information 3 Table S3: Harmonized data.

Supporting Information 4 Table S4: The phenoscanner result.

Supporting Information 5 Table S5: Outlier Summary.

Supporting Information 6 Figure S1: WBC_to_DEP_RadialMR.

Supporting Information 7 Table S6: The final IVs for WBC to DEP.

Supporting Information 8 Table S7: WBC_to_DEP MR results.

Supporting Information 9 Figure S2: WBC_to_DEP_scatter.

Supporting Information 10 Table S8: Extract IVs for depression.

Supporting Information 11 Table S9: Harmonized data.

Supporting Information 12 Table S10: The phenoscanner result.

Supporting Information 13 Table S11: Outlier Summary.

Supporting Information 14 Table S12: The final IVs for DEP to WBC.

Supporting Information 15 Table S13: DEP_to_WBC MR results.

Supporting Information 16 Figure S3: DEP_to_WBC_scatter.

Supporting Information 17 Table S14: WBC_to_DEP_heterogeneity.

Supporting Information 18 Table S15: DEP_to_WBC_heterogeneity.

Supporting Information 19 Table S16: WBC_to_DEP_pleiotropy_test.

Supporting Information 20 Table S17: DEP_to_WBC_pleiotropy_test.

Supporting Information 21 Table S18: WBC_to_DEP_MR_PRESSO.

Supporting Information 22 Table S19: DEP_to_WBC_MR_PRESSO.

Supporting Information 23 Figure S3: WBC_to_DEP_funnel plot.

Supporting Information 24 Figure S4: DEP_to_WBC_funnel plot.

Supporting Information 25 Figure S5: WBC_to_DEP_leaveone.

Supporting Information 26 Figure S7: DEP_to_WBC_leaveone.

Supporting Information 27 Table S20: The information of the data utilized in the article.
