# Supplementary material for: Revealing the Causal Relationship Between Differential White Blood Cell Counts and Depression: A Bidirectional Two-Sample Mendelian Randomization Study
Source: Depress Anxiety. 2025 Mar 3;2025:3131579. doi: 10.1155/da/3131579 (PMC11987073; doi:10.1155/da/3131579)
Supplement: Supporting Information 1 — Table S1: STROBE-MR checklist. [file 3131579.f1.docx]

Table S_2 The information of the data utilized in the article

| **Author(s)** | **year** | **trait** | **samplesize** | **URL** | **Database and Identifier** |
| --- | --- | --- | --- | --- | --- |
| Dragana Vuckovic ,et al | 2020 | basophil cell count | 563,946 | https://gwas.mrcieu.ac.uk/datasets/ieu-b-29/ | IEU OpenGWAS, ieu-b-29 |
| Dragana Vuckovic ,et al | 2020 | white blood cell count | 563,946 | https://gwas.mrcieu.ac.uk/datasets/ieu-b-30/ | IEU OpenGWAS, ieu-b-30 |
| Dragana Vuckovic ,et al | 2020 | monocyte cell count | 563,946 | https://gwas.mrcieu.ac.uk/datasets/ieu-b-31/ | IEU OpenGWAS, ieu-b-31 |
| Dragana Vuckovic ,et al | 2020 | lymphocyte cell count | 563,946 | https://gwas.mrcieu.ac.uk/datasets/ieu-b-32/ | IEU OpenGWAS, ieu-b-32 |
| Dragana Vuckovic ,et al | 2020 | eosinophil cell count | 563,946 | https://gwas.mrcieu.ac.uk/datasets/ieu-b-33/ | IEU OpenGWAS, ieu-b-33 |
| Dragana Vuckovic ,et al | 2020 | neutrophil cell count | 563,946 | https://gwas.mrcieu.ac.uk/datasets/ieu-b-34/ | IEU OpenGWAS, ieu-b-34 |
| FinnGen consortium | 2023 | depression | 406,986 | https://storage.googleapis.com/finngen-public-data-r10/summary_stats/finngen_R10_F5_DEPRESSIO.gz | documentation of R10 release, F5_DEPRESSIO |
