# Supplementary material for: Revealing the Causal Relationship Between Differential White Blood Cell Counts and Depression: A Bidirectional Two-Sample Mendelian Randomization Study
Source: Depress Anxiety. 2025 Mar 3;2025:3131579. doi: 10.1155/da/3131579 (PMC11987073; doi:10.1155/da/3131579)
Supplement: Supporting Information 5 — Table S5: Outlier Summary. [file 3131579.f5.pdf]

	finn					
	ieu-b-29	ieu-b-30	ieu-b-31	ieu-b-32	ieu-b-33	ieu-b-34
1. MR_PRESSO	rs7078507	rs61863767	rs3856364	rs10846577<br>rs9534338	rs62086903<br>rs73086541	rs10786325<br>rs9277764
2. RadialMR	rs10906375<br>rs118013485<br>rs12447180<br>rs1295927<br>rs1537061<br>rs16989483<br>rs2007774<br>rs310631<br>rs4911102<br>rs62105478<br>rs6543144<br>rs72721631<br>rs7613595<br>rs7684939	rs10102877<br>rs11993347<br>rs1228024<br>rs13248936<br>rs2104415<br>rs2665405<br>rs2729707<br>rs2875974<br>rs301817<br>rs3857488<br>rs41317014<br>rs4535497<br>rs60466842<br>rs62007171<br>rs62311395<br>rs6502608<br>rs6554195<br>rs6952262<br>rs696<br>rs7177<br>rs72973711<br>rs73028871<br>rs73191188<br>rs7326825<br>rs74679834<br>rs8084255<br>rs921313<br>rs9330650<br>rs9382100<br>rs9508005<br>rs9863<br>rs13291664	rs1091815<br>rs11086102<br>rs1127101<br>rs11844354<br>rs12332674<br>rs12480462<br>rs12742428<br>rs12756133<br>rs13032491<br>rs13271228<br>rs146039611<br>rs17498743<br>rs1902796<br>rs1930303<br>rs1970364<br>rs2063996<br>rs2176777<br>rs2302774<br>rs2665405<br>rs2810883<br>rs28367597<br>rs2846573<br>rs3128959<br>rs41381344<br>rs4411554<br>rs45577137<br>rs4907230<br>rs4970966<br>rs57221391<br>rs6772164<br>rs7196129<br>rs723585<br>rs73142138<br>rs73203055<br>rs745822<br>rs7569084<br>rs7593080<br>rs7824937<br>rs9787298<br>rs113292043<br>rs12542907<br>rs6796	rs10145277<br>rs1017875<br>rs11168249<br>rs12542907<br>rs12598978<br>rs1292069<br>rs139974673<br>rs1637366<br>rs1807669<br>rs1991431<br>rs2055101<br>rs2412544<br>rs2548256<br>rs2727487<br>rs2755253<br>rs34030812<br>rs34499378<br>rs35010780<br>rs3810818<br>rs4129560<br>rs4368798<br>rs4619033<br>rs4696314<br>rs4880192<br>rs55977949<br>rs58265751<br>rs62389639<br>rs6487543<br>rs6929523<br>rs7312770<br>rs74344617<br>rs7527389<br>rs77057307<br>rs7780328<br>rs7790229<br>rs78270096<br>rs7839516<br>rs78976959<br>rs8181326	rs10059018<br>rs10876550<br>rs11204682<br>rs118013485<br>rs12154498<br>rs13226583<br>rs140509806<br>rs159963<br>rs1672753<br>rs1689510<br>rs180506<br>rs2025489<br>rs2043293<br>rs2979489<br>rs34212866<br>rs34439695<br>rs3812206<br>rs3824867<br>rs3846855<br>rs4680250<br>rs4908835<br>rs4931002<br>rs56117721<br>rs62105489<br>rs62395833<br>rs62473720<br>rs637064<br>rs6556313<br>rs668248<br>rs6684992<br>rs72766638<br>rs73238201<br>rs73272842<br>rs7569084<br>rs79881201<br>rs8012643<br>rs8113367<br>rs925966<br>rs9840310<br>rs9939774	rs10252457<br>rs11580229<br>rs11625865<br>rs11690365<br>rs11993347<br>rs12189880<br>rs12487658<br>rs12658947<br>rs12909505<br>rs13291664<br>rs1411424<br>rs16961474<br>rs17728<br>rs207253<br>rs2421200<br>rs2665405<br>rs2729707<br>rs2955958<br>rs301817<br>rs3777755<br>rs3856364<br>rs4535497<br>rs609264<br>rs62011281<br>rs6468341<br>rs6511703<br>rs68002561<br>rs6855981<br>rs694180<br>rs723585<br>rs72973711<br>rs7322886<br>rs73271394<br>rs9265980<br>rs9508005<br>rs820384
