# Supplementary figures and images for: Revealing the Causal Relationship Between Differential White Blood Cell Counts and Depression: A Bidirectional Two-Sample Mendelian Randomization Study
Source: Depress Anxiety. 2025 Mar 3;2025:3131579. doi: 10.1155/da/3131579 (PMC11987073; doi:10.1155/da/3131579)

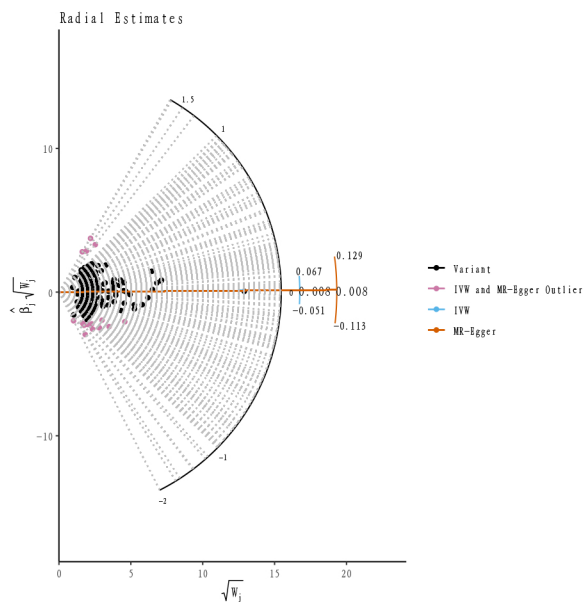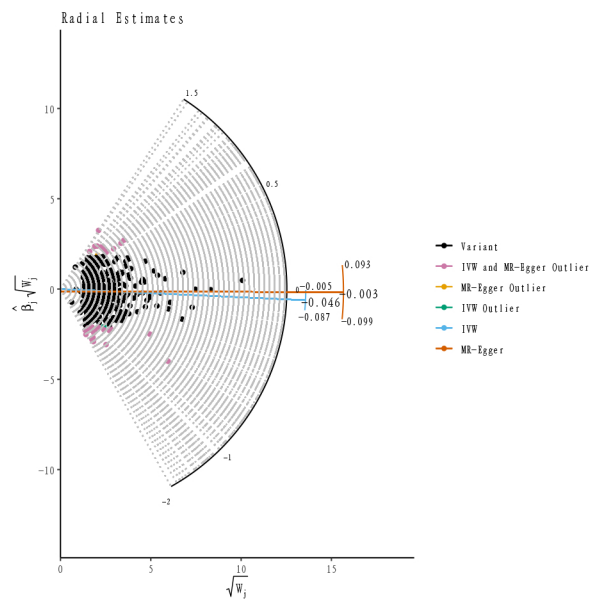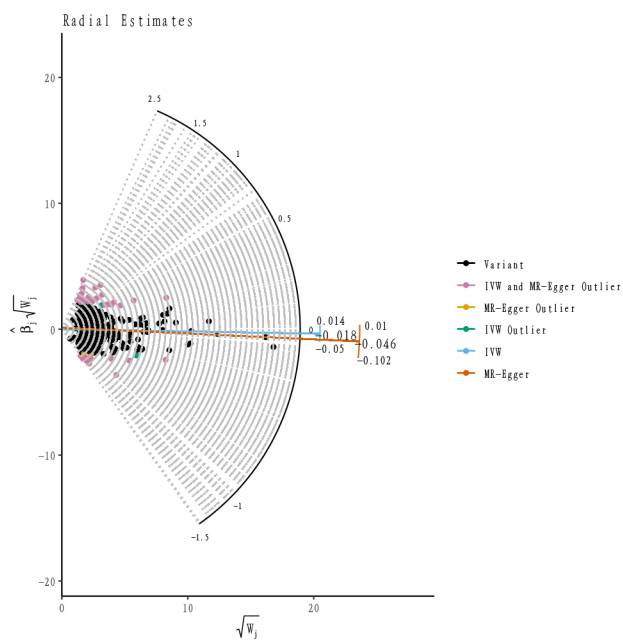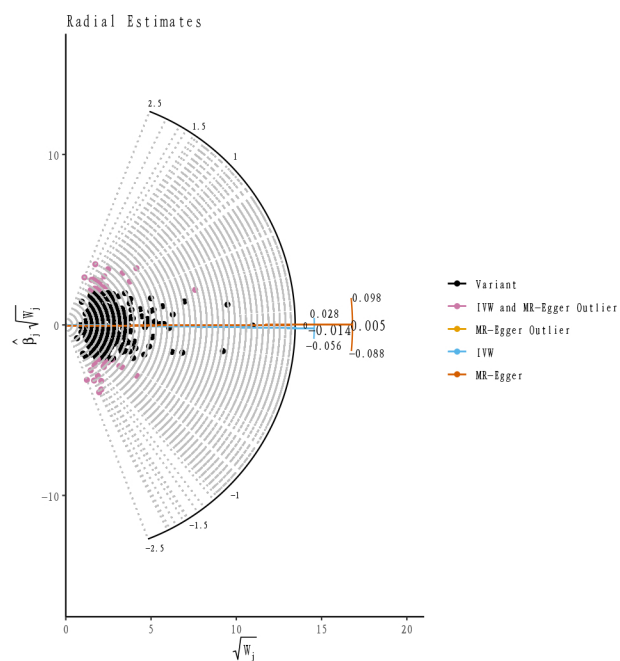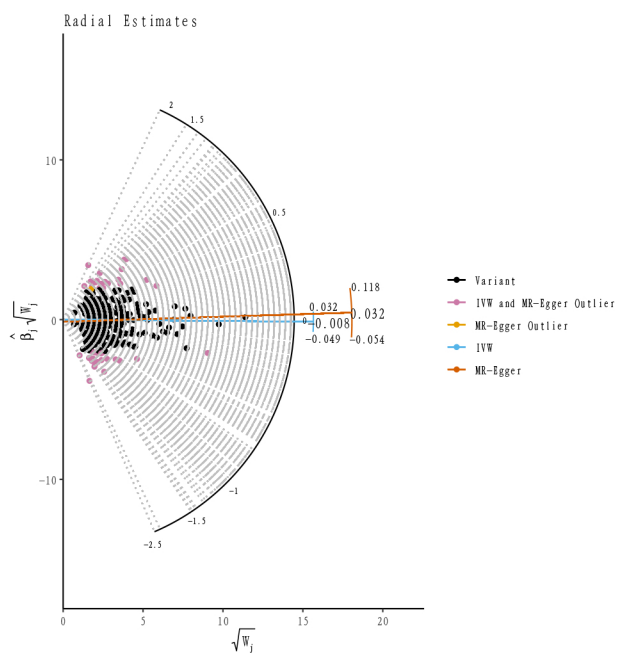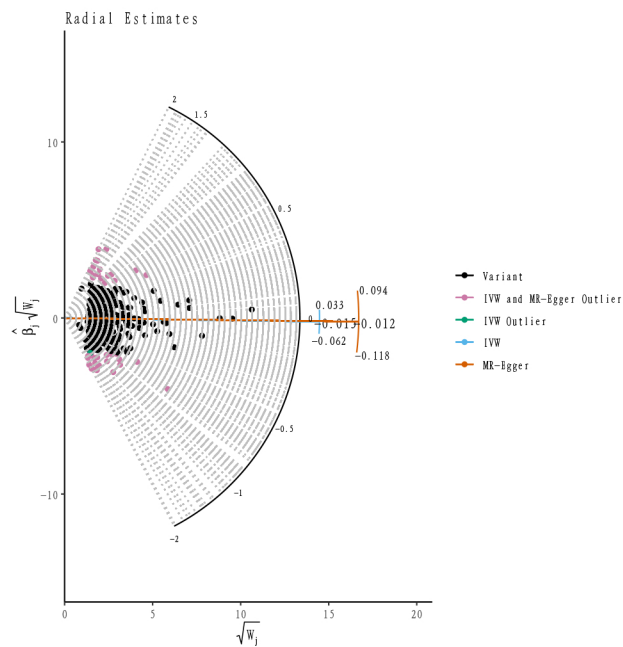

Supplement: Supporting Information 6 — Figure S1: WBC_to_DEP_RadialMR. [file 3131579.f6.pdf]

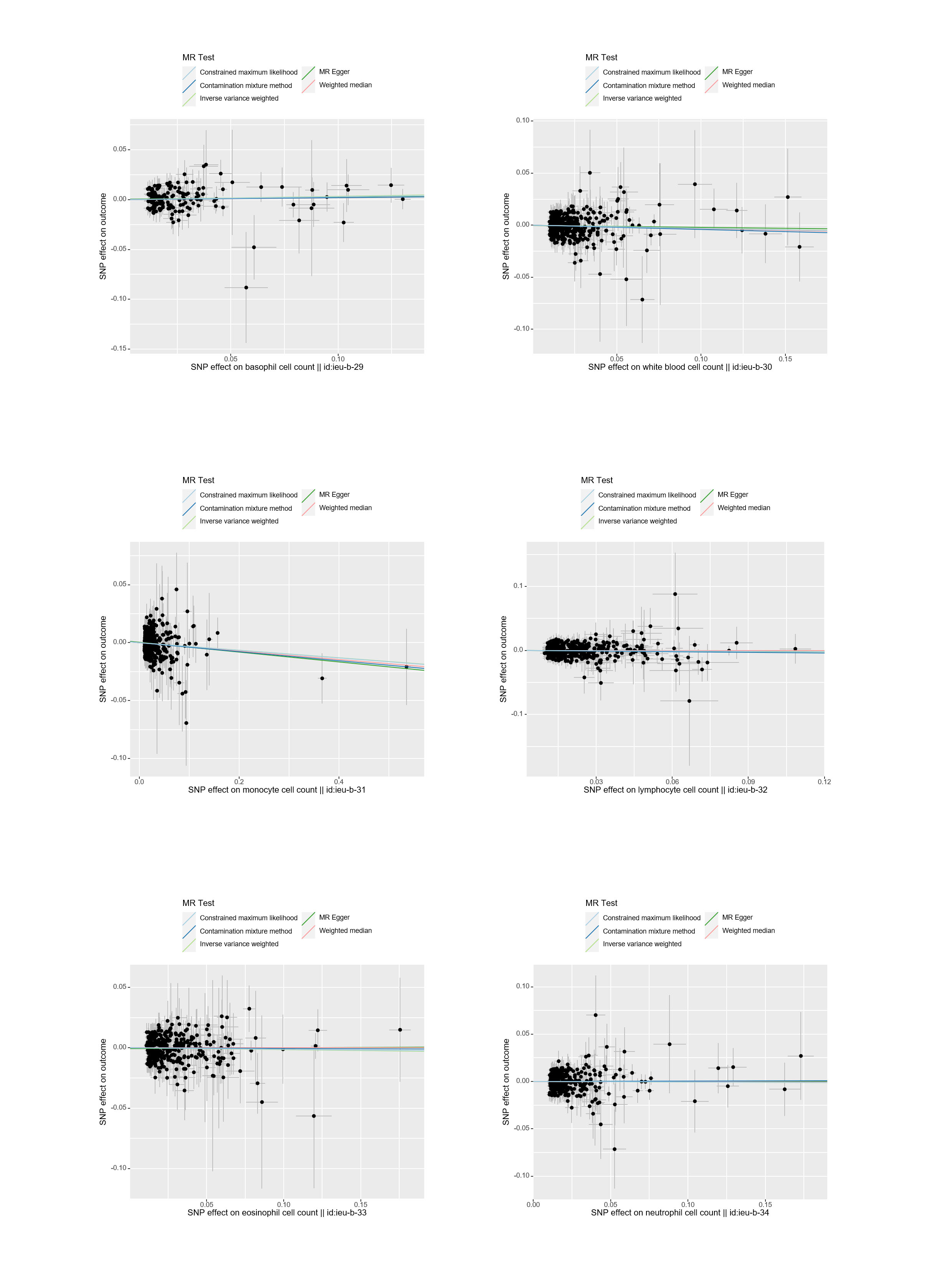

Supplement: Supporting Information 9 — Figure S2: WBC_to_DEP_scatter. [file 3131579.f9.jpg]

Radial Estimates

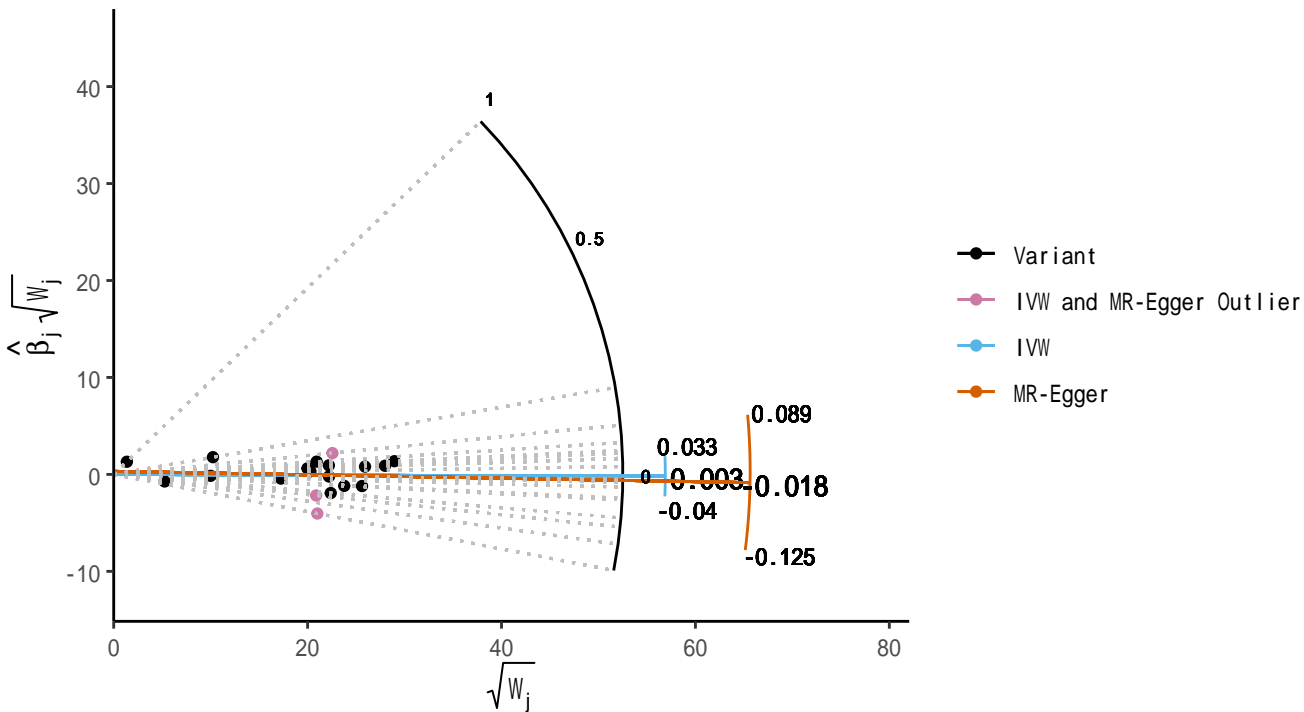

Supplement: Supporting Information 13 — Table S11: Outlier Summary. [file 3131579.f13.pdf]

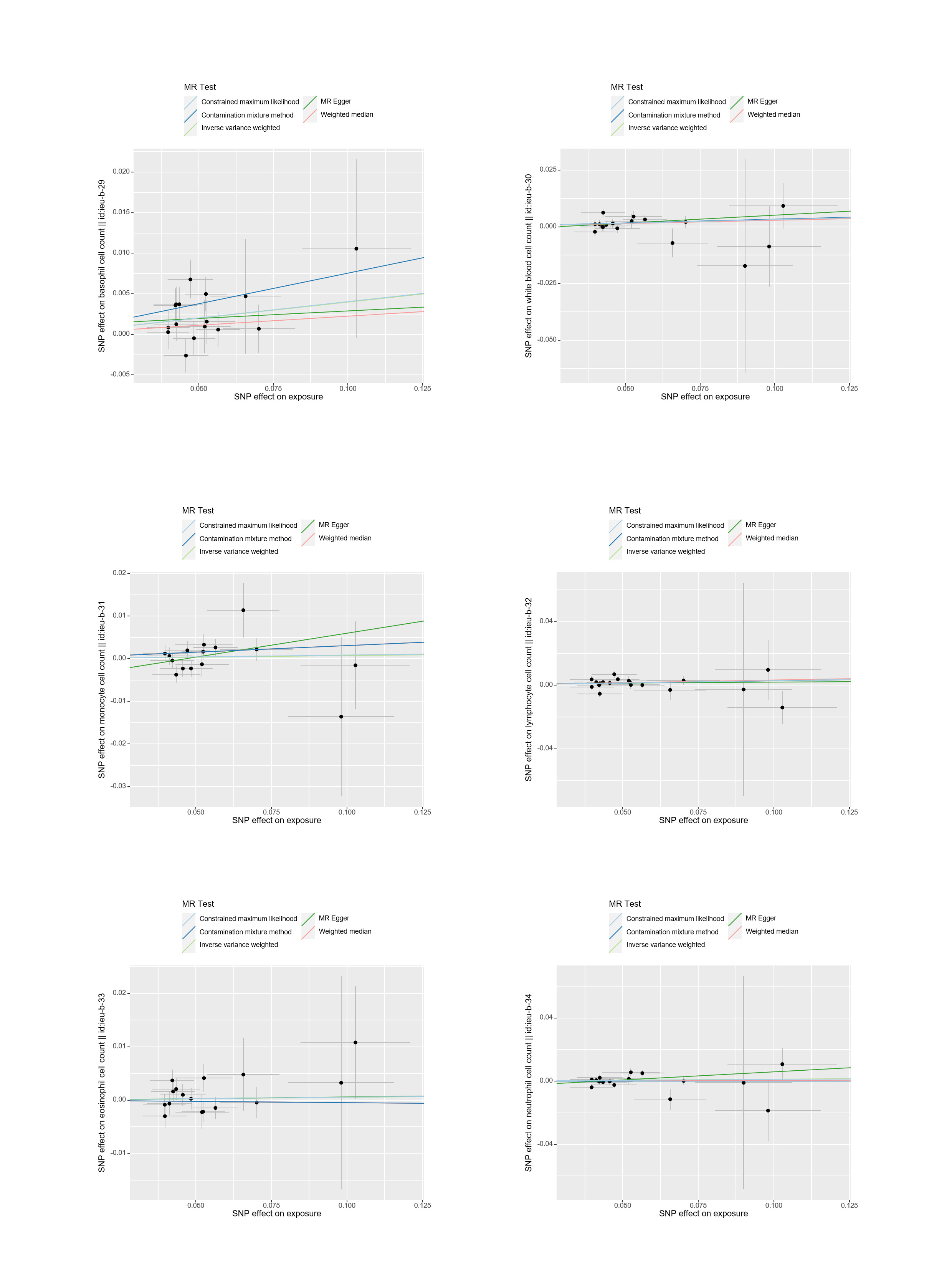

Supplement: Supporting Information 16 — Figure S3: DEP_to_WBC_scatter. [file 3131579.f16.jpg]

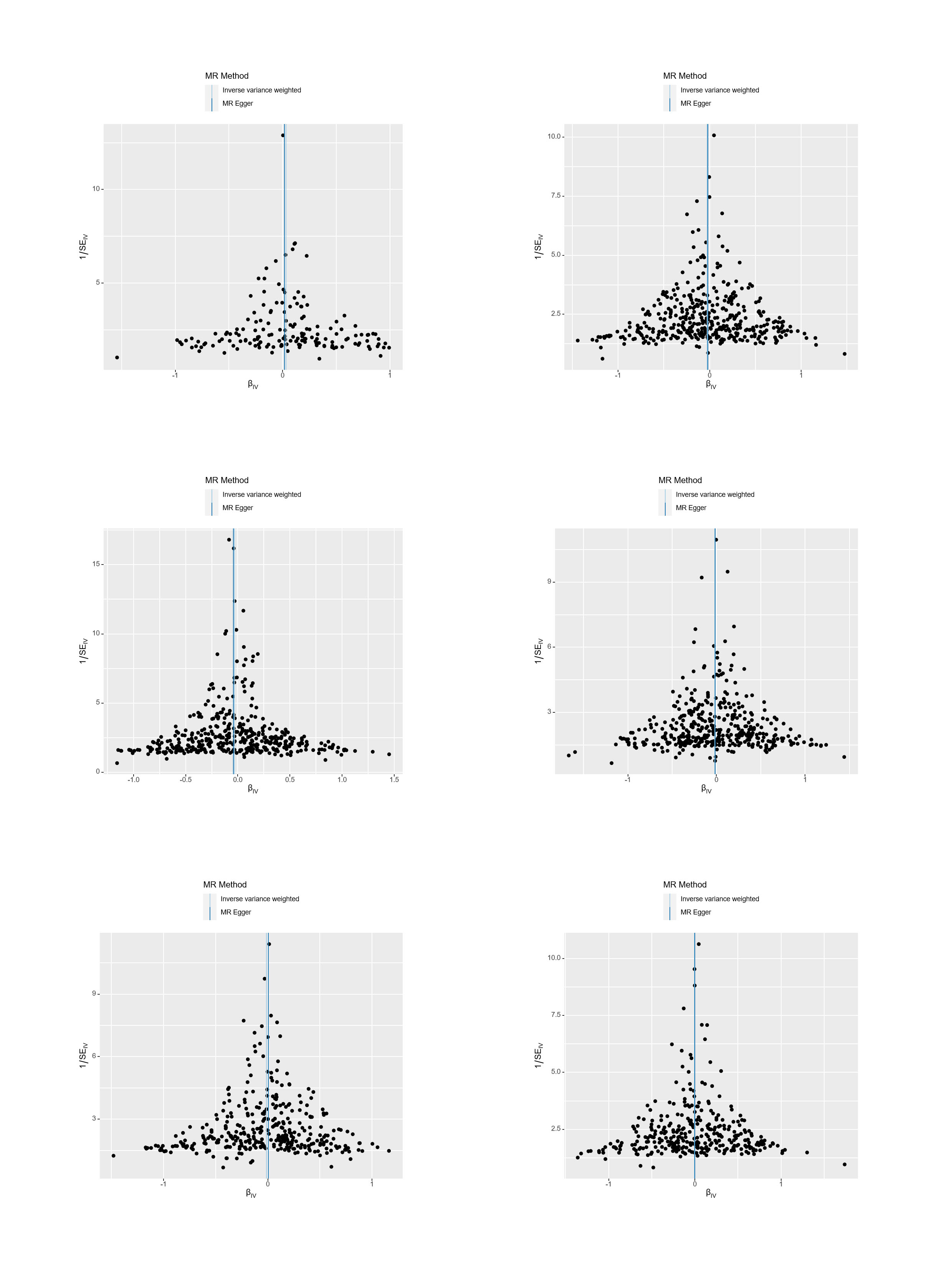

Supplement: Supporting Information 23 — Figure S3: WBC_to_DEP_funnel plot. [file 3131579.f23.jpg]

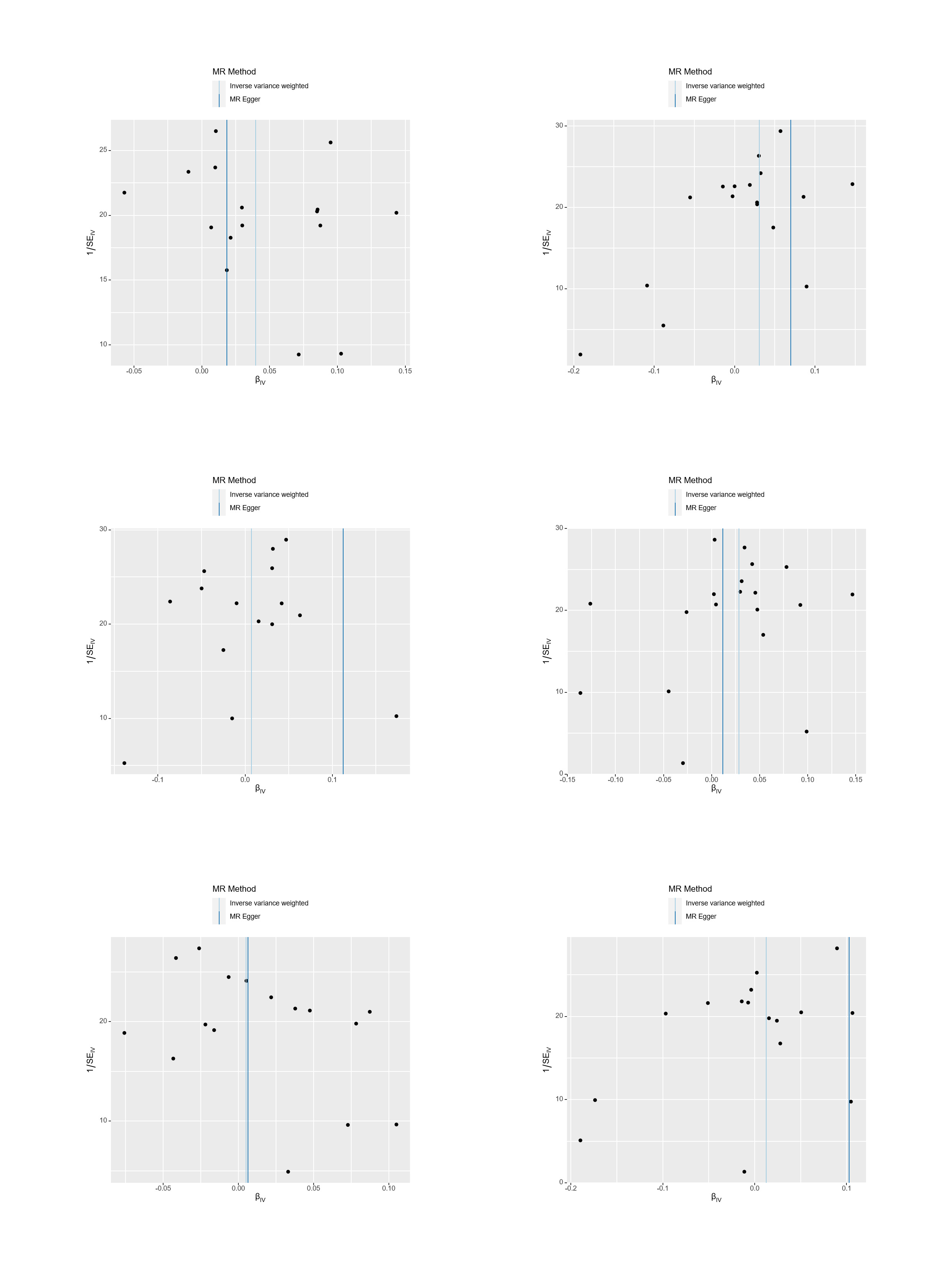

Supplement: Supporting Information 24 — Figure S4: DEP_to_WBC_funnel plot. [file 3131579.f24.jpg]

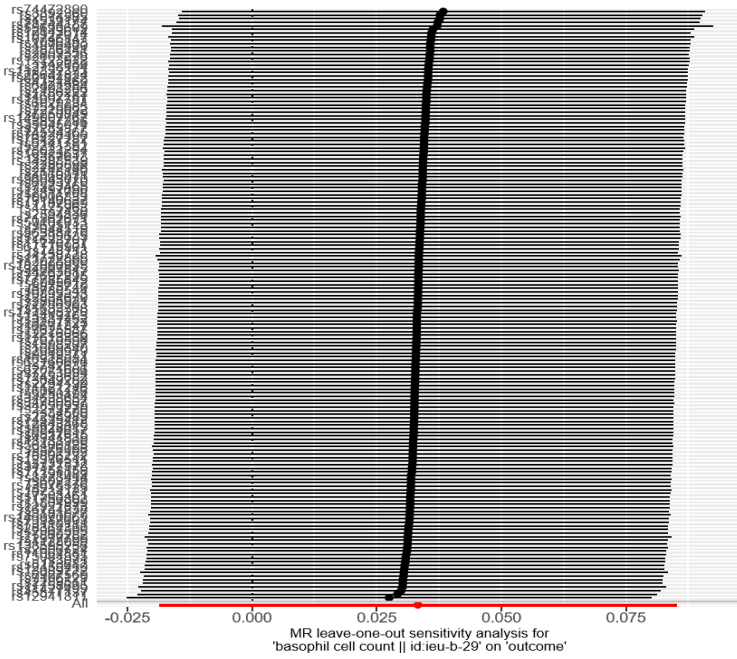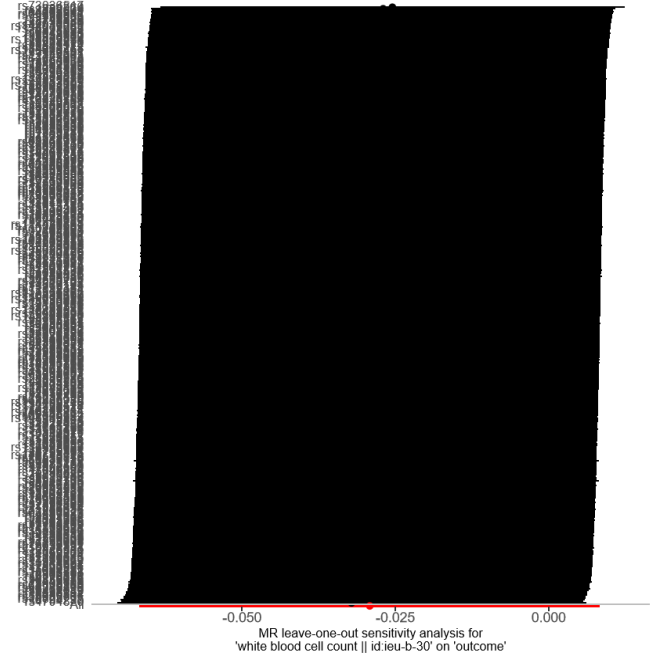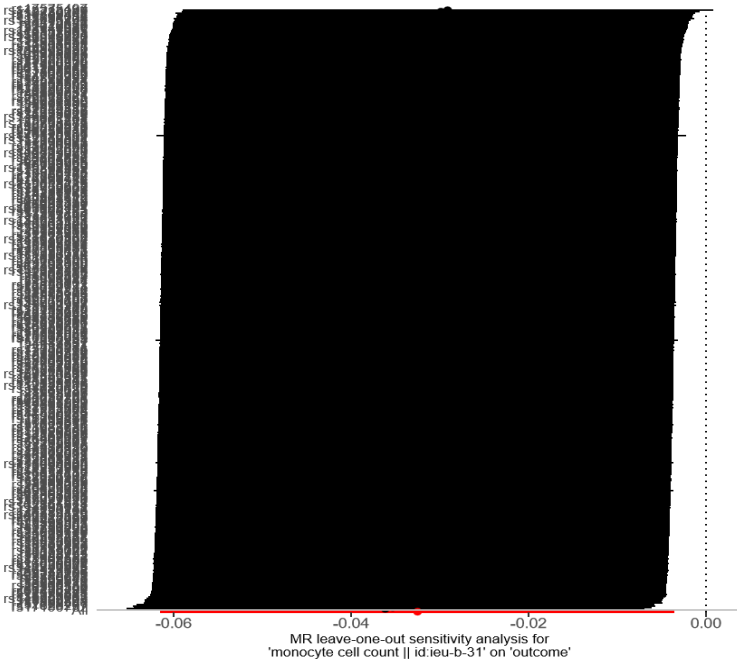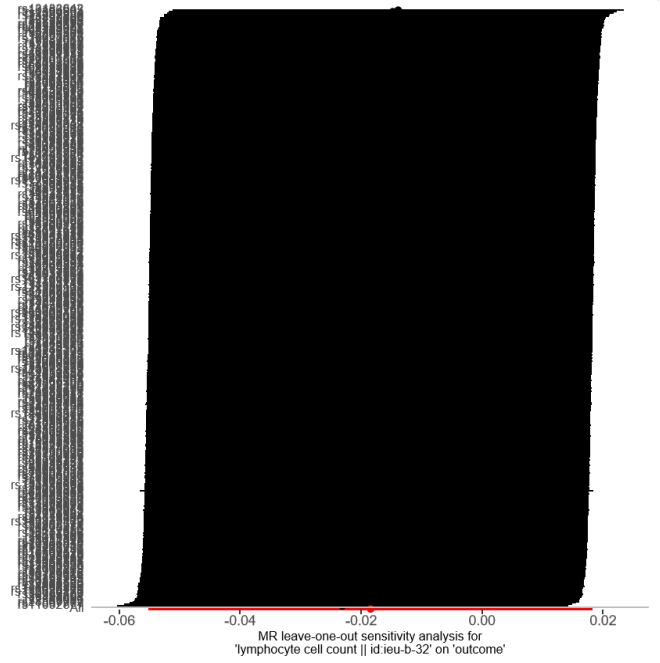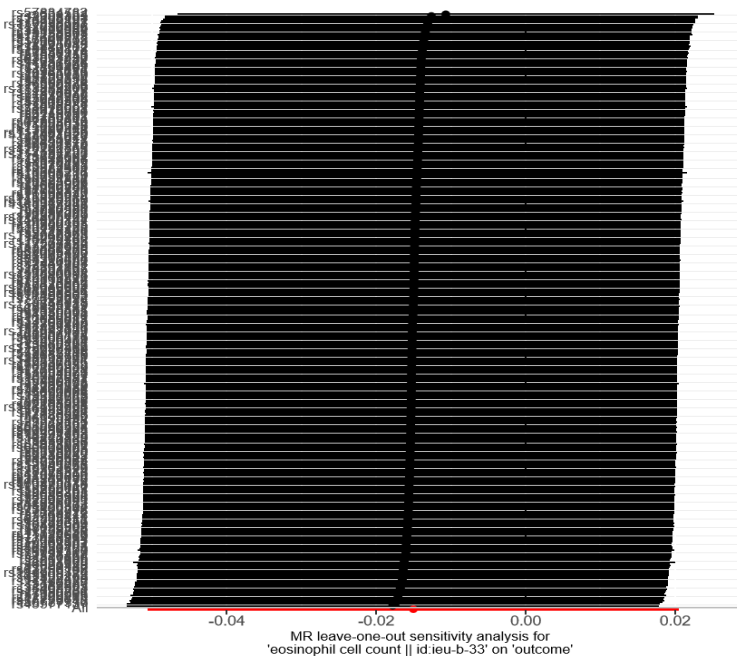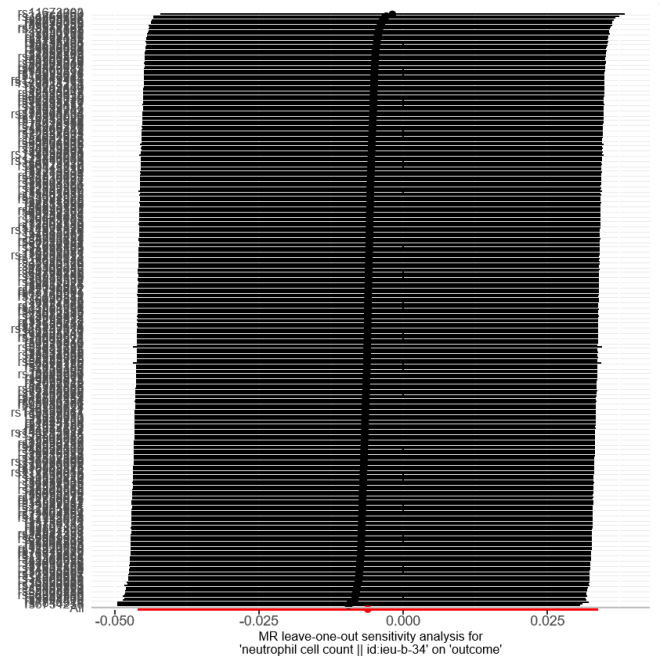

Supplement: Supporting Information 25 — Figure S5: WBC_to_DEP_leaveone. [file 3131579.f25.pdf]

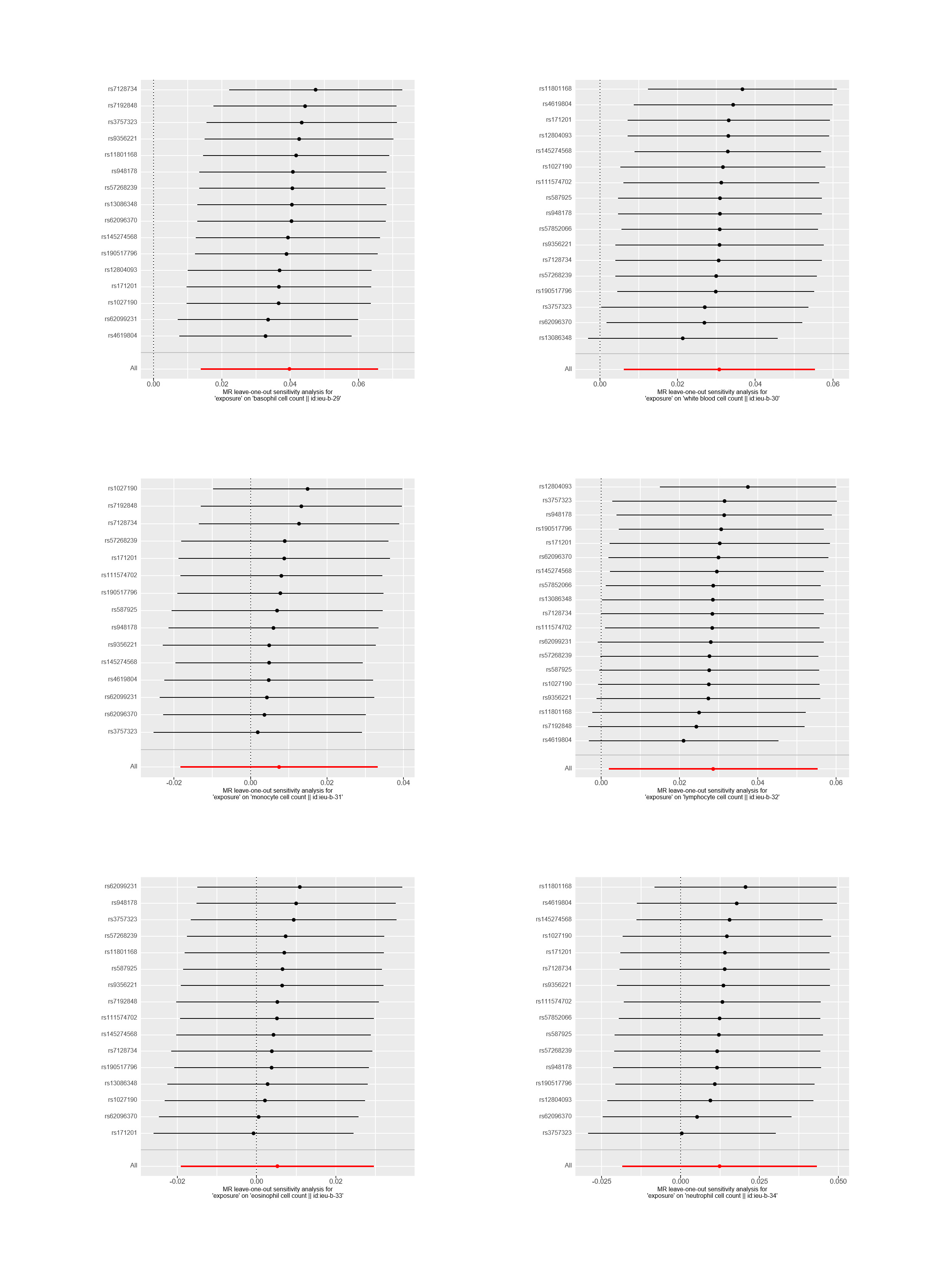

Supplement: Supporting Information 26 — Figure S7: DEP_to_WBC_leaveone. [file 3131579.f26.jpg]
