# Supplementary material for: Revealing the Causal Relationship Between Differential White Blood Cell Counts and Depression: A Bidirectional Two-Sample Mendelian Randomization Study
Source: Depress Anxiety. 2025 Mar 3;2025:3131579. doi: 10.1155/da/3131579 (PMC11987073; doi:10.1155/da/3131579)
Supplement: Supporting Information 8 — Table S7: WBC_to_DEP MR results. [file 3131579.f8.pdf]

exposure	outcome	method	nsnp	b	se	pval	or	or_lci95	or_uci95	FDR
basophil cel	finngen_DEPRESSIO	Inverse va	164	0.0332765	0.0265148	0.20947186	1.0338364	0.981481208	1.08898438	0.4189437
basophil cel	finngen_DEPRESSIO	MR Egger	164	0.017861	0.0470216	0.70455644	1.0180215	0.928391846	1.11630425	
basophil cel	finngen_DEPRESSIO	Weighted m	164	0.0192345	0.0445168	0.66568778	1.01942072	0.934243303	1.11236398	
basophil cel	finngen_DEPRESSIO	Contaminat	164	0.0198359	0.0331633	0.60711576	1.02003395	0.955840628	1.08853844	
basophil cel	finngen_DEPRESSIO	Constraine	164	0.0292727	0.030288	0.33380475	1.02970535	0.970356483	1.09268411	
white blood	finngen_DEPRESSIO	Inverse va	391	-0.029165	0.0191513	0.12779313	0.97125642	0.935474611	1.00840689	0.3833794
white blood	finngen_DEPRESSIO	MR Egger	391	-0.018046	0.0405402	0.65647345	0.98211619	0.907098286	1.06333814	
white blood	finngen_DEPRESSIO	Weighted m	391	-0.033134	0.0325668	0.30894902	0.96740852	0.907587489	1.03117248	
white blood	finngen_DEPRESSIO	Contaminat	391	-0.041264	0.0280612	0.1050789	0.95957606	0.908224491	1.01383108	
white blood	finngen_DEPRESSIO	Constraine	391	-0.030273	0.0196974	0.1243107	0.9701802	0.933438413	1.0083682	
monocyte cel	finngen_DEPRESSIO	Inverse va	411	-0.032501	0.0147858	0.02793836	0.96802108	0.94037027	0.99648494	0.1676302
monocyte cel	finngen_DEPRESSIO	MR Egger	411	-0.042517	0.024057	0.07791968	0.95837462	0.914234404	1.00464598	
monocyte cel	finngen_DEPRESSIO	Weighted m	411	-0.036293	0.0259665	0.16220601	0.9643575	0.916505109	1.01470835	
monocyte cel	finngen_DEPRESSIO	Contaminat	411	-0.038924	0.0178571	0.04798471	0.96182368	0.928742158	0.99608356	
monocyte cel	finngen_DEPRESSIO	Constraine	411	-0.032863	0.0159114	0.03888541	0.96767071	0.937958254	0.99832438	
lymphocyte c	finngen_DEPRESSIO	Inverse va	400	-0.018413	0.0187367	0.32575458	0.98175591	0.946355969	1.01848003	0.4885501
lymphocyte c	finngen_DEPRESSIO	MR Egger	400	-0.015096	0.0400069	0.70613204	0.98501769	0.910729582	1.06536547	
lymphocyte c	finngen_DEPRESSIO	Weighted m	400	-0.002033	0.0330573	0.95096876	0.99796936	0.935358947	1.06477076	
lymphocyte c	finngen_DEPRESSIO	Contaminat	400	-0.033444	0.0255102	0.45538225	0.96710942	0.919942938	1.01669418	
lymphocyte c	finngen_DEPRESSIO	Constraine	400	-0.015549	0.0198955	0.43447601	0.98457087	0.946916389	1.0237227	
eosinophil c	finngen_DEPRESSIO	Inverse va	352	-0.01503	0.0181308	0.40712509	0.98508263	0.950691105	1.02071828	0.4885501
eosinophil c	finngen_DEPRESSIO	MR Egger	352	0.0064315	0.036242	0.85925017	1.00645221	0.937439687	1.08054531	
eosinophil c	finngen_DEPRESSIO	Weighted m	352	0.0008746	0.0291258	0.97604372	1.00087501	0.945338674	1.05967397	
eosinophil c	finngen_DEPRESSIO	Contaminat	352	-0.005094	0.0306122	0.85992259	0.99491901	0.936979442	1.05644137	
eosinophil c	finngen_DEPRESSIO	Constraine	352	-0.012486	0.0188944	0.50873009	0.98759192	0.951687417	1.024851	
neutrophil c	finngen_DEPRESSIO	Inverse va	328	-0.006131	0.0203623	0.76336011	0.99388824	0.95500309	1.03435668	0.7633601
neutrophil c	finngen_DEPRESSIO	MR Egger	328	-0.001853	0.0406681	0.9636854	0.9981487	0.921675095	1.08096751	
neutrophil c	finngen_DEPRESSIO	Weighted m	328	-0.003196	0.0356895	0.92865302	0.99680947	0.929464152	1.06903438	
neutrophil c	finngen_DEPRESSIO	Contaminat	328	0.0041341	0.0306122	0.85964719	1.00414268	0.945665967	1.0662354	
neutrophil c	finngen_DEPRESSIO	Constraine	328	-0.00548	0.0206357	0.79057267	0.99453484	0.955112614	1.03558422	
