# Supplementary material for: Revealing the Causal Relationship Between Differential White Blood Cell Counts and Depression: A Bidirectional Two-Sample Mendelian Randomization Study
Source: Depress Anxiety. 2025 Mar 3;2025:3131579. doi: 10.1155/da/3131579 (PMC11987073; doi:10.1155/da/3131579)
Supplement: Supporting Information 12 — Table S10: The phenoscanner result. [file 3131579.f12.pdf]

exposure	outcome	methord	outlier
finngen_DEPRESSION	ieu-b-29	MR_PRESSO	rs587925
		Funnel plot	rs111574702 rs57852066
	ieu-b-30	MR_PRESSO	rs62099231 rs7192848
	ieu-b-31	MR_PRESSO	rs12804093
		RadialMR	rs11801168 rs13086348
		Funnel plot	rs57852066
	ieu-b-32	MR_PRESSO	NA
	ieu-b-33	MR_PRESSO	NA
		Funnel plot	rs57852066
	ieu-b-34	MR_PRESSO	rs13086348 rs62099231 rs7192848
