# Supplementary material for: Revealing the Causal Relationship Between Differential White Blood Cell Counts and Depression: A Bidirectional Two-Sample Mendelian Randomization Study
Source: Depress Anxiety. 2025 Mar 3;2025:3131579. doi: 10.1155/da/3131579 (PMC11987073; doi:10.1155/da/3131579)
Supplement: Supporting Information 15 — Table S13: DEP_to_WBC MR results. [file 3131579.f15.pdf]

exposure	outcome	method	nsnp	b	se	pval	lo_ci	up_ci	FDR
finngen_DEPRESSIO	basophil cell count	Inverse variance	16	0.0398253	0.0132323	0.002615	0.01389	0.0657606	0.0156901
		MR Egger	16	0.0185472	0.0847677	0.8299632	-0.147598	0.1846919	
		Weighted median	16	0.0224302	0.0181299	0.2160134	-0.013104	0.0579647	
		Contamination mix	16	0.0754838	0.0357143	0.0639561	0.0054838	0.1454838	
		Constrained maxin	16	0.0403791	0.0144732	0.0052721	0.0120116	0.0687467	
finngen_DEPRESSIO	white blood cell count	Inverse variance	17	0.0307355	0.0125713	0.0144893	0.0060958	0.0553752	0.04346798
		MR Egger	17	0.0699419	0.0732724	0.3549381	-0.073672	0.2135558	
		Weighted median	17	0.0286429	0.0167195	0.0866855	-0.004127	0.0614131	
		Contamination mix	17	0.0335039	0.0127551	0.0741214	0.0085039	0.0585039	
		Constrained maxin	17	0.0316915	0.0128822	0.0138901	0.0064423	0.0569407	
finngen_DEPRESSIO	monocyte cell count	Inverse variance	15	0.0074592	0.013174	0.5712515	-0.018362	0.0332802	0.67315005
		MR Egger	15	0.1123366	0.0777452	0.1721469	-0.040044	0.2647171	
		Weighted median	15	0.0309599	0.0175142	0.0771096	-0.003368	0.0652877	
		Contamination mix	15	0.0308868	0.0178571	0.1489364	-0.004113	0.0658868	
		Constrained maxin	15	0.0087099	0.0123086	0.4791795	-0.015415	0.0328348	
finngen_DEPRESSIO	lymphocyte cell count	Inverse variance	19	0.0286051	0.0136116	0.0355952	0.0019263	0.0552839	0.07119038
		MR Egger	19	0.0116169	0.0859846	0.8941161	-0.156913	0.1801467	
		Weighted median	19	0.033036	0.0152706	0.0305126	0.0031056	0.0629663	
		Contamination mix	19	0.0283159	0.0127551	0.0312203	0.0033159	0.0533159	
		Constrained maxin	19	0.0291945	0.0138392	0.0348963	0.0020698	0.0563193	
finngen_DEPRESSIO	eosinophil cell count	Inverse variance	16	0.0052521	0.0124509	0.6731501	-0.019152	0.0296559	0.67315005
		MR Egger	16	0.0064945	0.0728431	0.9302203	-0.136278	0.1492669	
		Weighted median	16	-0.004882	0.0167902	0.7712534	-0.03779	0.0280273	
		Contamination mix	16	-0.004792	0.0357143	1	-0.074792	0.0652084	
		Constrained maxin	16	0.0054424	0.0140171	0.697818	-0.022031	0.032916	
finngen_DEPRESSIO	neutrophil cell count	Inverse variance	16	0.0123771	0.0157447	0.4318026	-0.018483	0.0432367	0.64770392
		MR Egger	16	0.1025575	0.0872217	0.259273	-0.068397	0.273512	
		Weighted median	16	0.0049151	0.0192944	0.7989241	-0.032902	0.0427321	
		Contamination mix	16	-0.000119	0.0204082	1	-0.040119	0.0398812	
		Constrained maxin	16	0.0111066	0.0151365	0.4630929	-0.018561	0.0407743	
