# Supplementary material for: Revealing the Causal Relationship Between Differential White Blood Cell Counts and Depression: A Bidirectional Two-Sample Mendelian Randomization Study
Source: Depress Anxiety. 2025 Mar 3;2025:3131579. doi: 10.1155/da/3131579 (PMC11987073; doi:10.1155/da/3131579)
Supplement: Supporting Information 17 — Table S14: WBC_to_DEP_heterogeneity. [file 3131579.f17.pdf]

exposure	outcome	method	Q	Q_df	Q_pval
finngen_DEPRESSION	basophil cell count	MR Egger	21.14064217	16	0.173167572
		Inverse variance weighted	21.56812032	17	0.201903455
finngen_DEPRESSION	white blood cell count	MR Egger	17.25919213	15	0.303607497
		Inverse variance weighted	17.59908835	16	0.347890116
finngen_DEPRESSION	monocyte cell count	MR Egger	15.91660591	14	0.318493577
		Inverse variance weighted	18.15174944	15	0.254771717
finngen_DEPRESSION	lymphocyte cell count	MR Egger	26.6108226	17	0.064027866
		Inverse variance weighted	26.67358723	18	0.085324607
finngen_DEPRESSION	eosinophil cell count	MR Egger	14.48567146	15	0.489058716
		Inverse variance weighted	14.48611678	16	0.562547473
finngen_DEPRESSION	neutrophil cell count	MR Egger	20.35173501	14	0.119436305
		Inverse variance weighted	21.9576964	15	0.108917963
