# Supplementary material for: Revealing the Causal Relationship Between Differential White Blood Cell Counts and Depression: A Bidirectional Two-Sample Mendelian Randomization Study
Source: Depress Anxiety. 2025 Mar 3;2025:3131579. doi: 10.1155/da/3131579 (PMC11987073; doi:10.1155/da/3131579)
Supplement: Supporting Information 18 — Table S15: DEP_to_WBC_heterogeneity. [file 3131579.f18.pdf]

exposure	outcome	method	Q	Q_df	Q_pval
basophil cell count	finngen_DEPRESSIO	MR Egger	138.4632	162	0.9097775
		Inverse variance weighted	138.62078	163	0.9172338
white blood cell count	finngen_DEPRESSIO	MR Egger	346.90396	389	0.9386147
		Inverse variance weighted	347.0008	390	0.9424923
monocyte cell count	finngen_DEPRESSIO	MR Egger	342.34892	409	0.9927961
		Inverse variance weighted	342.62745	410	0.9932815
lymphocyte cell count	finngen_DEPRESSIO	MR Egger	339.16486	398	0.9851498
		Inverse variance weighted	339.17367	399	0.9864514
eosinophil cell count	finngen_DEPRESSIO	MR Egger	300.82175	350	0.9730866
		Inverse variance weighted	301.28946	351	0.9742581
neutrophil cell count	finngen_DEPRESSIO	MR Egger	293.49923	326	0.9017236
		Inverse variance weighted	293.51399	327	0.9083523
