# Supplementary material for: Revealing the Causal Relationship Between Differential White Blood Cell Counts and Depression: A Bidirectional Two-Sample Mendelian Randomization Study
Source: Depress Anxiety. 2025 Mar 3;2025:3131579. doi: 10.1155/da/3131579 (PMC11987073; doi:10.1155/da/3131579)
Supplement: Supporting Information 19 — Table S16: WBC_to_DEP_pleiotropy_test. [file 3131579.f19.pdf]

exposure	outcome	egger_intercept	se	pval
basophil cell count	finngen_DEPRESSION	0.000483172	0.001217153	0.69191257
white blood cell count	finngen_DEPRESSION	-0.000287344	0.000923385	0.755826456
monocyte cell count	finngen_DEPRESSION	0.000375785	0.000712044	0.597955523
lymphocyte cell count	finngen_DEPRESSION	-8.76E-05	0.000933406	0.925287455
eosinophil cell count	finngen_DEPRESSION	-0.000659574	0.000964436	0.494493443
neutrophil cell count	finngen_DEPRESSION	-0.000114531	0.000942572	0.90336303
