# Supplementary material for: Revealing the Causal Relationship Between Differential White Blood Cell Counts and Depression: A Bidirectional Two-Sample Mendelian Randomization Study
Source: Depress Anxiety. 2025 Mar 3;2025:3131579. doi: 10.1155/da/3131579 (PMC11987073; doi:10.1155/da/3131579)
Supplement: Supporting Information 20 — Table S17: DEP_to_WBC_pleiotropy_test. [file 3131579.f20.pdf]

outcome	exposure	egger_intercept	se	pval
finngen_DEPRESSION	basophil cell count	0.002382457	0.004188584	0.577390962
finngen_DEPRESSION	white blood cell count	-0.001876003	0.003451636	0.594763927
finngen_DEPRESSION	monocyte cell count	-0.005433486	0.003875137	0.18265556
finngen_DEPRESSION	lymphocyte cell count	0.000820248	0.004096306	0.843668063
finngen_DEPRESSION	eosinophil cell count	7.38E-05	0.00349824	0.983441897
finngen_DEPRESSION	neutrophil cell count	-0.004358259	0.004146505	0.311032422
