# Supplementary material for: Revealing the Causal Relationship Between Differential White Blood Cell Counts and Depression: A Bidirectional Two-Sample Mendelian Randomization Study
Source: Depress Anxiety. 2025 Mar 3;2025:3131579. doi: 10.1155/da/3131579 (PMC11987073; doi:10.1155/da/3131579)
Supplement: Supporting Information 21 — Table S18: WBC_to_DEP_MR_PRESSO. [file 3131579.f21.pdf]

exposure	outcome	Causal Estimate	Sd	pval
basophil cell count	finngen_DEPRESSION	0.033276544	0.024451656	0.924
white blood cell count	finngen_DEPRESSION	-0.029164764	0.018064699	0.948
monocyte cell count	finngen_DEPRESSION	-0.032501413	0.01351649	0.9936667
lymphocyte cell count	finngen_DEPRESSION	-0.01841257	0.017274982	0.986
eosinophil cell count	finngen_DEPRESSION	-0.015029753	0.016797895	0.9766667
neutrophil cell count	finngen_DEPRESSION	-0.006130517	0.019291598	0.9073333
