# Supplementary material for: Revealing the Causal Relationship Between Differential White Blood Cell Counts and Depression: A Bidirectional Two-Sample Mendelian Randomization Study
Source: Depress Anxiety. 2025 Mar 3;2025:3131579. doi: 10.1155/da/3131579 (PMC11987073; doi:10.1155/da/3131579)
Supplement: Supporting Information 22 — Table S19: DEP_to_WBC_MR_PRESSO. [file 3131579.f22.pdf]

exposure	outcome	Causal Estimate	Sd	pval
finngen_DEPRESSION	basophil cell count	0.038251017	0.013993559	0.202
finngen_DEPRESSION	white blood cell count	0.030735475	0.012571273	0.3553333
finngen_DEPRESSION	monocyte cell count	0.007718434	0.013357709	0.2753333
finngen_DEPRESSION	lymphocyte cell count	0.028605112	0.013611618	0.09466667
finngen_DEPRESSION	eosinophil cell count	0.005070915	0.011845774	0.5626667
finngen_DEPRESSION	neutrophil cell count	0.01237708	0.015744702	0.112
