# Supplementary material for: Revealing the Causal Relationship Between Differential White Blood Cell Counts and Depression: A Bidirectional Two-Sample Mendelian Randomization Study
Source: Depress Anxiety. 2025 Mar 3;2025:3131579. doi: 10.1155/da/3131579 (PMC11987073; doi:10.1155/da/3131579)
Supplement: Supporting Information 27 — Table S20: The information of the data utilized in the article. [file 3131579.f27.docx]

**Table S1 STROBE-MR checklist of recommended items to address in reports of Mendelian randomization studies**^1^ ^2^

| **Item No.** | **Section** | **Checklist item** | **Note/ line No.** |
| --- | --- | --- | --- |
| 1 | **TITLE and ABSTRACT** | Indicate Mendelian randomization (MR) as the study’s design in the title and/or the abstract if that is a main purpose of the study | ‘Mendelian randomization’ in title and abstract |
|  | **INTRODUCTION** |  |  |
| 2 | **Background** | Explain the scientific background and rationale for the reported study. What is the exposure? Is a potential causal relationship between exposure and outcome plausible? Justify why MR is a helpful method to address the study question | This study investigates the potential causal relationship between white blood cell counts (exposure) and depression (outcome) using Mendelian randomization analysis, as traditional observational studies have shown inconsistent results regarding their association, and MR can help overcome limitations of confounding and reverse causality while providing stronger evidence for causality through genetic instruments [Introduction, paragraphs 1-7]. |
| 3 | **Objectives** | State specific objectives clearly, including pre-specified causal hypotheses (if any). State that MR is a method that, under specific assumptions, intends to estimate causal effects | This study aims to investigate the bidirectional causal relationships between white blood cell counts (including subtypes) and depression using Mendelian randomization analysis, which, under specific genetic instrumental assumptions, can estimate causal effects. This represents the first comprehensive MR study examining these relationships, extending beyond previous research that was limited to specific WBC subtypes [Introduction, final paragraph]. |
|  | **METHODS** |  |  |
| 4 | **Study design and data sources** | Present key elements of the study design early in the article. Consider including a table listing sources of data for all phases of the study. For each data source contributing to the analysis, describe the following: | 1. Setting:This is a two-sample Mendelian Randomization study utilizing summary statistics from large-scale GWAS data. The study design is detailed in Methods section 2.1, including the rigorous selection of instrumental variables and the bidirectional MR analysis framework. 2. Participants: The WBC data[Methods 2.2.1] and depression data [Methods 2.2.2] 3. Genetic variants: Methods 2.3.1 4. Assessment methods: WBC traits [Methods 2.2.1] and depression diagnosis [Methods 2.2.2]. 5. Ethics: The study utilized publicly available data that had been previously collected and documented, making additional ethical approval unnecessary [as noted in the final paragraph of the Methods section] |
|  | a) | Setting: Describe the study design and the underlying population, if possible. Describe the setting, locations, and relevant dates, including periods of recruitment, exposure, follow-up, and data collection, when available. |  |
|  | b) | Participants: Give the eligibility criteria, and the sources and methods of selection of participants. Report the sample size, and whether any power or sample size calculations were carried out prior to the main analysis |  |
|  | c) | Describe measurement, quality control and selection of genetic variants |  |
|  | d) | For each exposure, outcome, and other relevant variables, describe methods of assessment and diagnostic criteria for diseases |  |
|  | e) | Provide details of ethics committee approval and participant informed consent, if relevant |  |
| 5 | **Assumptions** | Explicitly state the three core IV assumptions for the main analysis (relevance, independence and exclusion restriction) as well assumptions for any additional or sensitivity analysis | The study adheres to the three fundamental assumptions of Mendelian Randomization (MR) for instrumental variables (IVs), as explicitly stated in Methods 2.1 and Figure 1 |
| 6 | **Statistical methods: main analysis** | Describe statistical methods and statistics used | a) Handling of Quantitative Variables [Methods 2.2.1 & 2.2.2]  b) Handling of Genetic Variants [Methods 2.3.1]  c) MR Estimator and Statistics [Methods 2.3.2]  d) Missing Data Handling: UK Biobank depression data were intentionally excluded to avoid sample overlap. Quality control measures ensured data completeness for included variants[Methods 2.2.2]  e) Multiple Testing [Methods 2.3.1 & 2.3.2] |
|  | a) | Describe how quantitative variables were handled in the analyses (i.e., scale, units, model) |  |
|  | b) | Describe how genetic variants were handled in the analyses and, if applicable, how their weights were selected |  |
|  | c) | Describe the MR estimator (e.g. two-stage least squares, Wald ratio) and related statistics. Detail the included covariates and, in case of two-sample MR, whether the same covariate set was used for adjustment in the two samples |  |
|  | d) | Explain how missing data were addressed |  |
|  | e) | If applicable, indicate how multiple testing was addressed |  |
| 7 | **Assessment of assumptions** | Describe any methods or prior knowledge used to assess the assumptions or justify their validity | Core IV Assumptions Assessment  [Methods 2.3.1] |
| 8 | **Sensitivity analyses and additional analyses** | Describe any sensitivity analyses or additional analyses performed (e.g. comparison of effect estimates from different approaches, independent replication, bias analytic techniques, validation of instruments, simulations) | We conducted sensitivity analyses using MR-Egger, MR-PRESSO, and weighted median methods to assess pleiotropy and robustness. Additional analyses included bidirectional MR testing for reverse causation with multiple testing corrections applied[Methods 2.3.2]. |
| 9 | **Software and pre-registration** |  |  |
|  | a) | Name statistical software and package(s), including version and settings used | 1. Software: [Methods 2.3.2, para 1, sent 1] 2. The research protocol and information are not registered |
|  | b) | State whether the study protocol and details were pre-registered (as well as when and where) |  |
|  | **RESULTS** |  |  |
| 10 | **Descriptive data** |  |  |
|  | a) | Report the numbers of individuals at each stage of included studies and reasons for exclusion. Consider use of a flow diagram | Not applicable, since in this study we used genome-wide summary statistics from previously published genome-wide association studies. We cite these in the main text. There is no sample overlap between the exposure and outcome studies |
|  | b) | Report summary statistics for phenotypic exposure(s), outcome(s), and other relevant variables (e.g. means, SDs, proportions) |  |
|  | c) | If the data sources include meta-analyses of previous studies, provide the assessments of heterogeneity across these studies |  |
|  | d) | For two-sample MR:   1. Provide justification of the similarity of the genetic variant-exposure associations between the exposure and outcome samples 2. Provide information on the number of individuals who overlap between the exposure and outcome studies |  |
| 11 | **Main results** |  |  |
|  | a) | Report the associations between genetic variant and exposure, and between genetic variant and outcome, preferably on an interpretable scale | a) Genetic Variants: [Results 3.1, para 1]  b) MR Estimates:  Forward MR (WBC → Depression): [Results 3.1, para 2]  Reverse MR (Depression → WBC): [Results 3.2]  c) No absolute risk calculations were reported in the manuscript.  d) Visualization:  Figure2 Figure3  Supporting information:Table S7, Figure S2, Table S13, Figure S3, |
|  | b) | Report MR estimates of the relationship between exposure and outcome, and the measures of uncertainty from the MR analysis, on an interpretable scale, such as odds ratio or relative risk per SD difference |  |
|  | c) | If relevant, consider translating estimates of relative risk into absolute risk for a meaningful time period |  |
|  | d) | Consider plots to visualize results (e.g. forest plot, scatterplot of associations between genetic variants and outcome versus between genetic variants and exposure) |  |
| 12 | **Assessment of assumptions** |  |  |
|  | a) | Report the assessment of the validity of the assumptions | Main text: [3.3 Sensitivity analysis]  Supporting information: Table S14, S15, S16, S17, S18, S19. |
|  | b) | Report any additional statistics (e.g., assessments of heterogeneity across genetic variants, such as *I^2^*, Q statistic or E-value) |  |
| 13 | **Sensitivity analyses and additional analyses** |  |  |
|  | a) | Report any sensitivity analyses to assess the robustnes  s of the main results to violations of the assumptions | a) Robustness Assessment: [Results 3.3]  b) Additional Analyses:  F-statistics assessment to evaluate weak instrument bias: [Results 3.1, para 1][Results 3.2, para 1]  Outlier detection and removal using MR-PRESSO and Radial MR: [ Supporting information:Table S5, Figure S1]  SNP validation through phenoscanner database: [ Supporting information: Table S4, S10]  Exclusion of confounding SNPs associated with depression risk factors  c) Bidirectional Analysis:  Forward MR (WBC → Depression): [Results 3.1, para 2, Supporting information Figure S2]  Reverse MR (Depression → WBC): [Results 3.2, Supporting information Figure S3]  d) Comparison with Non-MR Studies:[Introduction]  e) Visualization [Supporting information Figure S5, S7] |
|  | b) | Report results from other sensitivity analyses or additional analyses |  |
|  | c) | Report any assessment of direction of causal relationship (e.g., bidirectional MR) |  |
|  | d) | When relevant, report and compare with estimates from non-MR analyses |  |
|  | e) | Consider additional plots to visualize results (e.g., leave-one-out analyses) |  |
|  | **DISCUSSION** |  |  |
| 14 | **Key results** | Summarize key results with reference to study objectives | Forward MR analysis suggests monocytes may reduce depression risk  Reverse MR indicates depression may lead to increased WBC and basophil counts [Discussion Para 1] |
| 15 | **Limitations** | Discuss limitations of the study, taking into account the validity of the IV assumptions, other sources of potential bias, and imprecision. Discuss both direction and magnitude of any potential bias and any efforts to address them | [Discussion Para 5] |
| 16 | **Interpretation** |  |  |
|  | a) | Meaning: Give a cautious overall interpretation of results in the context of their limitations and in comparison with other studies | 1. Meaning:This pioneering study employs two-sample Mendelian randomization on a large-scale GWAS dataset, featuring a sample size 3.3 times larger than previous research, to investigate WBC-depression causality. Our findings both validate existing knowledge and contribute new insights to the field.[Discussion Para 4] 2. Mechanism:[Discussion Para 2] 3. Clinical relevance: Results offer insights for exploring depression risk factors.[Conclusions] |
|  | b) | Mechanism: Discuss underlying biological mechanisms that could drive a potential causal relationship between the investigated exposure and the outcome, and whether the gene-environment equivalence assumption is reasonable. Use causal language carefully, clarifying that IV estimates may provide causal effects only under certain assumptions |  |
|  | c) | Clinical relevance: Discuss whether the results have clinical or public policy relevance, and to what extent they inform effect sizes of possible interventions |  |
| 17 | **Generalizability** | Discuss the generalizability of the study results (a) to other populations, (b) across other exposure periods/timings, and (c) across other levels of exposure | Currently only generalizable to European populations[Discussion Para 5] |
|  | **OTHER INFORMATION** |  |  |
| 18 | **Funding** | Describe sources of funding and the role of funders in the present study and, if applicable, sources of funding for the databases and original study or studies on which the present study is based | Funding |
| 19 | **Data and data sharing** | Provide the data used to perform all analyses or report where and how the data can be accessed, and reference these sources in the article. Provide the statistical code needed to reproduce the results in the article, or report whether the code is publicly accessible and if so, where | Main text: Availability of data and materials,  Data Availability Statement, Supporting information Table S20.  The statistical code used in the article is not public, please ask the author for the review if needed. |
| 20 | **Conflicts of Interest** | All authors should declare all potential conflicts of interest | The authors declare that there is no conflict of interest. |

This checklist is copyrighted by the Equator Network under the Creative Commons Attribution 3.0 Unported (CC BY 3.0) license.

1. Skrivankova VW, Richmond RC, Woolf BAR, Yarmolinsky J, Davies NM, Swanson SA, et al. Strengthening the Reporting of Observational Studies in Epidemiology using Mendelian Randomization (STROBE-MR) Statement. JAMA. 2021;under review.

2. Skrivankova VW, Richmond RC, Woolf BAR, Davies NM, Swanson SA, VanderWeele TJ, et al. Strengthening the Reporting of Observational Studies in Epidemiology using Mendelian Randomisation (STROBE-MR): Explanation and Elaboration. BMJ. 2021;375:n2233.
